# Prospects and Trends in Biomedical Microelectromechanical Systems (MEMS) Devices: A Review

**DOI:** 10.3390/biom15060898

**Published:** 2025-06-18

**Authors:** Lowell Welburn, Amir Milad Moshref Javadi, Luong Nguyen, Salil Desai

**Affiliations:** 1 Department of Mechanical Engineering, North Carolina Agricultural and Technical State University, Greensboro, NC 27411, USA; lwelburn@aggies.ncat.edu; 2 Department of Industrial and Systems Engineering, North Carolina Agricultural and Technical State University, Greensboro, NC 27411, USA; amjavadi@aggies.ncat.edu (A.M.M.J.); ltnguyen@aggies.ncat.edu (L.N.)

**Keywords:** MEMS, NEMS, BioMEMS, biomedical, nanotechnology, biosensor

## Abstract

Designing and manufacturing devices at the micro- and nanoscales offers significant advantages, including high precision, quick response times, high energy density ratios, and low production costs. These benefits have driven extensive research in micro-electromechanical systems (MEMS) and nano-electromechanical systems (NEMS), resulting in various classifications of materials and manufacturing techniques, which are ultimately used to produce different classifications of MEMS devices. The current work aims to systematically organize the literature on MEMS in biomedical devices, encompassing past achievements, present developments, and future prospects. This paper reviews the current research trends, highlighting significant material advancements and emerging technologies in biomedical MEMS in order to meet the current challenges facing the field, such as ensuring biocompatibility, achieving miniaturization, and maintaining precise control in biological environments. It also explores projected applications, including use in advanced diagnostic tools, targeted drug delivery systems, and innovative therapeutic devices. By mapping out these trends and prospects, this review will help identify current research gaps in the biomedical MEMS field. By pinpointing these gaps, researchers can focus on addressing unmet needs and advancing state-of-the-art biomedical MEMS technology. Ultimately, this can lead to the development of more effective and innovative biomedical devices, improving patient care and outcomes.

## 1. Introduction

**Figure 1:**
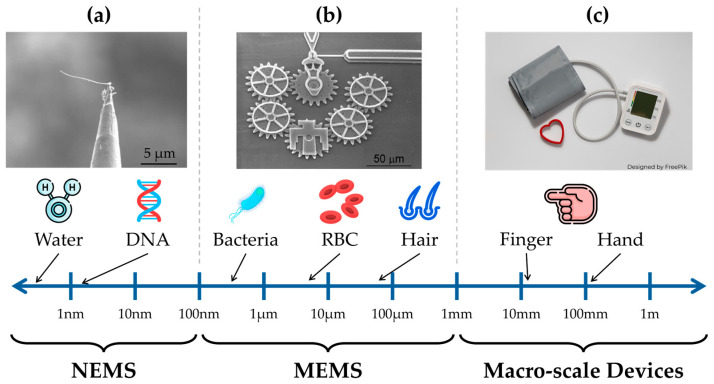
Examples of macro-, micro-, and nanoscale devices: (**a**) carbon nanotube for nanoswitch applications (reprinted with permission from Elsevier) [5]; (**b**) six-gear chain (reprinted with permission from Elsevier) [6]; (**c**) blood pressure monitor.

Since the birth of transistors in the 1940s, there has been an explosion of technologies leveraging the advantages of working at the micro- and nanoscale [1]. The popularization of the silicon substrates used to micromachine integrated circuits (IC’s) made room for the introduction of microelectromechanical systems (MEMS) devices [2]. Loosely speaking, MEMS devices are devices that combine electrical and mechanical components into one unified device, with characteristic sizes spanning from 1 mm to 100 nm [2]. Figure 1 illustrates some examples of features and devices at various nano-, micro-, and macroscales. These devices enable a certain task to be accomplished while also meeting miniaturization criteria including (1) the use of multiple components, (2) the ability to be mass-produced, (3) system integration, and (4) complex functions [2]. Smaller devices ranging from 100 nm to 1 nm are often referred to as nano-electromechanical systems (NEMS). Although the scale of many devices presented in this review reaches the nanoscale, these devices will be referred to as MEMS devices to simplify the discussion and match the bulk of the literature. MEMS devices are becoming essential parts of patient monitoring, treatment, and diagnosis, as medical device precision and downsizing continue to progress using different materials and manufacturing methods [3,4].

The choice of material in fabricating MEMS devices is an active research area, especially in biomedical applications where challenges arise regarding biocompatibility and durability. Currently, the vast majority of MEMS are fabricated using silicon, polymer, metal, piezoelectric, and 2D materials. There are also a wide array of common MEMS device categories, such as sensors, actuators, microfluidics, RF, electromagnetics, energy harvesters, thermo-tensile, and optical devices. For each application, there are unique challenges and solutions that will be addressed in detail.

The manufacturing process implemented when producing MEMS devices varies enormously depending on the final application. Methods such as additive manufacturing (also known as the bonding technique), surface micromachining, and bulk micromachining are a few common examples that will be discussed later in this text.

The biomedical applications of MEMS devices are practically limitless. The purpose of this review is to provide a roadmap to newcomers and veterans in the field of biomedical MEMS devices. This will allow for the continued progression of biomedical MEMS by identifying potential research gaps and opportunities for study.

## 2. Materials and Categories of MEMS Devices

**Figure 2:**
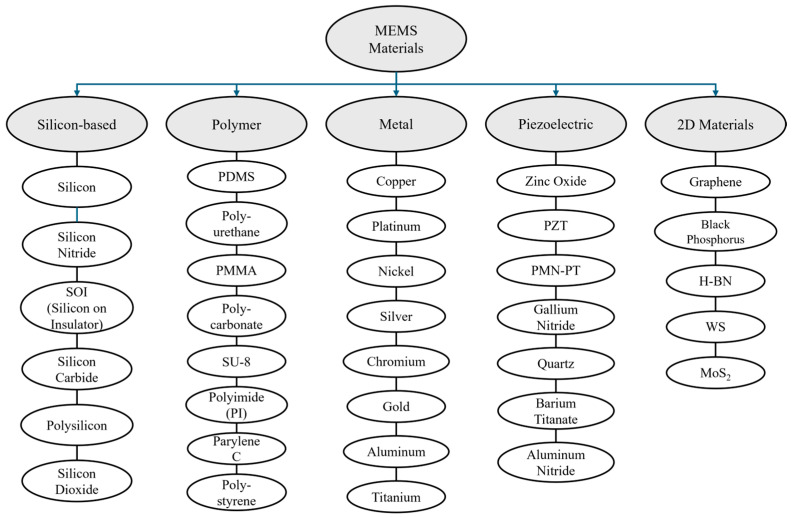
Common MEMS materials.

NEMS and MEMS are innovative gadgets used in a wide range of applications, such as sensors, actuators, medical devices, and communication systems. The specific categories, materials, classifications, components, and operating principles that control the functioning of these gadgets dictate the level of performance of each device [7]. Figure 2 presents an overview of common materials used in the manufacturing of MEMS devices. The unique characteristics and advantages of these materials are leveraged depending on the specific application that the MEMS device will be implemented within.

### 2.1. Materials Used in MEMS Devices

#### 2.1.1. Silicon

The advancements in silicon photolithographic technology, starting in the 1960s, have served as the backbone for the development of MEMS [8]. Silicon is the most widely used material for MEMS devices due to its unique mechanical properties, availability, and compatibility with complementary metal–oxide semiconductor (CMOS)-processing technology. It can be used for many different applications; for example, single-crystal silicon (SCS) can be used for structural components and silicon dioxide can be used as an insulating layer. In many cases silicon, can be implemented within high-precision sensors and actuators [9,10]. As a material for MEMS devices, silicon carbide has garnered a lot of interest because it is chemically inert, has a higher mechanical strength, and is more thermally stable than traditional silicon, especially in harsh environmental conditions [11]. Notwithstanding issues like brittleness and heat sensitivity, silicon is the fundamental material propelling advances in MEMS technology in a variety of industries, such as consumer electronics, automotive, and healthcare, due to its compatibility with the current semiconductor processes and its resilience under a wide range of circumstances [12].

#### 2.1.2. Polymers

**Figure 3:**
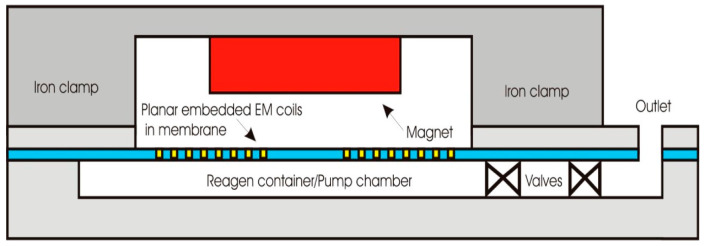
Schematic of a PDMS-based electromagnetic micropump and valve system with embedded planar microcoils and a magnetic diaphragm (reprinted from open access) [15].

In terms of cost, mechanical characteristics, and processing simplicity, polymer materials provide numerous benefits. Because of this, many MEMS sensors and actuators use polymer materials, such as fibers, plastics, and elastomers. The research and development on polymeric polymers have made it possible to create extremely flexible structures and substrates with unique bulk and surface properties using different cost-effective manufacturing techniques. Recent major advancements in polymer micromachining, such as deposition, removal, and release processes for three commonly used MEMS polymer materials (SU-8, polyimide, and parylene C), have allowed for the successful evolution of polymers within MEMS [13]. Biomedical and flexible MEMS devices often use polyimide and polydimethylsiloxane (PDMS) because of their biocompatibility and adaptability, making them a popular choice for wearable and lab-on-a-chip applications [14]. A PDMS-based electromagnetic micropump with embedded planar coils and a magnetically driven diaphragm, as seen in Figure 3, is a commonly used design example [15]. Because of its chemical and thermal resilience, polyimide is also frequently utilized in flexible electronics and sensors [14]. Both PDMS and PMMA are extremely hydrophobic, and unlike silicon, their adhesive force is unaffected by their rest time or relative humidity according to contact angle measurements and Laplace force computations [16]. Nanocomposite elastomers are another category of material that are often utilized because they combine elasticity, strength, and enhanced functionality through the integration of nanoparticles, resulting in improved mechanical and electrical properties [17,18]. MEMS uses polystyrene with nanoparticles to create inexpensive microfluidics with improved optical and functional characteristics [19]. Gold is used as the adhesive layer and electrode to spin-coat a silicon substrate with pure polyurethane and nanocomposite carbon black (CB) polyurethane solutions [20]. Polymers are also being researched for use in circuitry, memory, and displays. However, adding polymers to MEMS devices for structural or functional reasons presents new problems, such as inadequate thermal stability, challenges in attaining precise dimensional control, and incompatibilities with conventional MEMS manufacturing techniques and fabrication processes. In MEMS, polymers have a variety of functions. For example, PDMS is utilized for flexible substrates and microfluidics, SU-8 and polyimide function as structural materials, and parylene C is used in protective coatings. Additionally, several polymer composites are being explored as sensing or active layers [17]. Another example of polymers is polycarbonate which, because of its superior transparency, biocompatibility, and ease of microfabrication, particularly in biomedical and microfluidic applications, is frequently utilized as a thermoplastic polymer in MEMS [21].

#### 2.1.3. Metals

Metals with superior electrical and mechanical conductivity, such as aluminum, nickel, and gold, are commonly utilized in MEMS. When creating metal MEMS, electrodeposition is a crucial step. Electrodeposition is the process in which, under the influence of an applied electric field, metal ions from a solution are electrochemically deposited onto a conductive substrate to produce thin metallic films or structures. Nickel is commonly utilized in electroplated MEMS components due to its durability, while gold is favored in applications requiring corrosion resistance and biocompatibility, such as biomedical sensors [22]. Research has examined the use of electrodeposited Ni-Cu alloys and nanocomposites as potential nickel substitutes for microsystems. In MEMS, conductivity is essential for sensing, actuation, and signal transfer. Conductive polymers are commonly utilized to provide lightweight, flexible substitutes for metals [23]. Aluminum microheaters and thin-film nickel temperature sensors are integrated into glass substrates using MEMS fabrication techniques for low-temperature sensor and actuator applications. Accurate resistance-based temperature calibration is made easier through the improved accuracy and linear performance of these components, which are usually enclosed within a PDMS membrane. Environmental and biological sensing applications that require accurate heat control at microscales benefit greatly from this strategy [24]. The benefits of high accuracy and high structural strength can also be combined using a hybrid process that merges the silicon-based and metal-based processes [25]. Metal sputtering, electroplating, and (inductively coupled plasma) ICP etching are the primary components of this technique.

#### 2.1.4. Piezoelectric

Piezoelectric devices are made of materials that, when subjected to mechanical stress, generate electric charges. On the other hand, when exposed to an electric field, these materials undergo deformation that allows for precise control over mechanical displacement. Typical piezoelectric materials, such as lead zirconate titanate (PZT), aluminum nitride (AlN), quartz, and zinc oxide (ZnO), are widely utilized in MEMS sensors, energy harvesters, accelerometers, ultrasonic transducers, and actuators due to the fact that they can transform mechanical strain into electrical energy and vice versa [26,27,28,29]. Because of their high piezoelectric coefficients, ferroelectric PZT-based perovskite thin films are being extensively researched for the creation of small piezoelectric energy harvesting (EH) power MEMS [30]. Piezoelectric materials have applications in several large-scale devices, including mobile phones and ultrasonic imaging [26]. Piezoelectric resonators and pressure sensors are also used in automotive and industrial applications, and can serve as crucial components of timing devices [26,31].

#### 2.1.5. Two-Dimensional Materials

Devices based on the use of two-dimensional materials for MEMS and NEMS exhibit distinctive features and increased sensitivity in comparison to their silicon-based equivalents [32]. It has been shown that 2D materials are viable building blocks for future electronics, and their mechanical qualities are crucial for a number of applications [33]. Graphene and other 2D materials with exceptional electrical, mechanical, and thermal properties, such as molybdenum disulfide (MoS_2_), have attracted a lot of attention recently. Because of its superior mechanical strength and conductivity, graphene is employed in MEMS and is a promising material for next-generation sensors and electronics [34,35,36]. The main issues with these materials are the lack of experimental data at low temperatures and the uneven application of high strain rates. Some researchers have created devices based on MEMS to address these issues by characterizing semiconductor materials and 2D nanomaterials at low temperatures [37]. The need for NEMS devices and sensing based on 2D materials is increased due to their unique features. High-performing mass sensors, gas sensors, accelerometers, pressure sensors, and microphones have been developed in recent decades utilizing suspended 2D membranes coupled with MEMS and NEMS [32].

### 2.2. Categories of MEMS Devices

**Figure 4:**
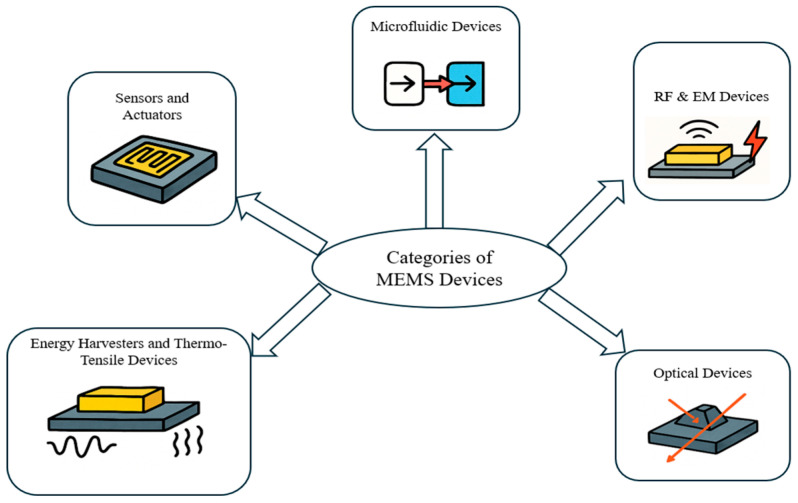
MEMS device categories.

Microelectromechanical systems (MEMS) are classified based on their functional applications. Figure 4 presents key categories, such as sensors, actuators, RF components, optical systems, and microfluidics. Each type is briefly explained in the following sections, with emphasis on their relevance in fields like biomechanics and biomedical engineering.

#### 2.2.1. Sensors and Actuators

MEMS sensors have been utilized in several industries, including consumer electronics, the automotive industry, and the medical industry. Devices such as biosensors, gyroscopes, accelerometers, and pressure sensors fall under this umbrella. Due to their diminutive stature, fine precision, and minimal power use, MEMS sensors are highly prized and essential to systems like those found in automobiles and smartphones [38,39]. As fifth-generation cellular network technology (5G) continues to quickly advance, MEMS and NEMS sensors will play an increasingly important role in providing information from 5G phones in daily life [40].

**Figure 5:**
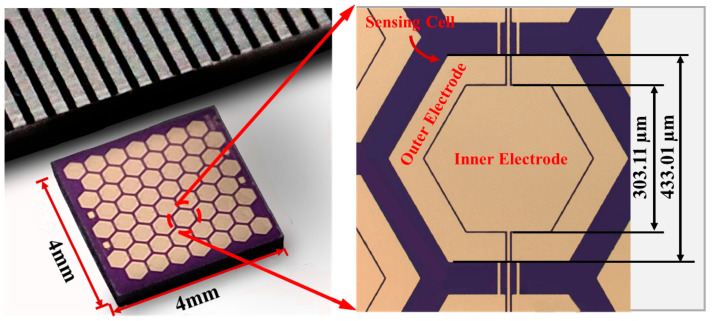
A 4 × 4 mm piezoelectric honeycomb MEMS diaphragm for hydrophone arrays. The honeycomb pattern increases sensitivity and structural uniformity (reprinted from open access) [43].

MEMS actuators, which convert various forms of energy into microscopic mechanical movements, are making a growing contribution and opening new capabilities in the fields of biomedical engineering and healthcare. Before making an informed decision as to which MEMS actuator is best for a given application out of the many types in the MEMS industry, the intended use and the characteristics of the actuators in question must be determined [4]. Generally, these actuators are divided into groups according to various driving actuation principles, including thermal, piezoelectric, and electrostatic principles. For example, piezoelectric actuators are employed in energy harvesters and microfluidics, while electrostatic actuators are frequently found in optical MEMS devices like micromirrors [41,42]. MEMS-based acoustic sensors, such as hydrophones, are becoming significant in underwater and biomedical sensing applications because of their sensitivity and small size. An MEMS hydrophone for acoustic pressure sensing with a honeycomb diaphragm and PDMS-encapsulated packaging is shown in Figure 5 [43].

#### 2.2.2. Microfluidic Devices

**Figure 6:**
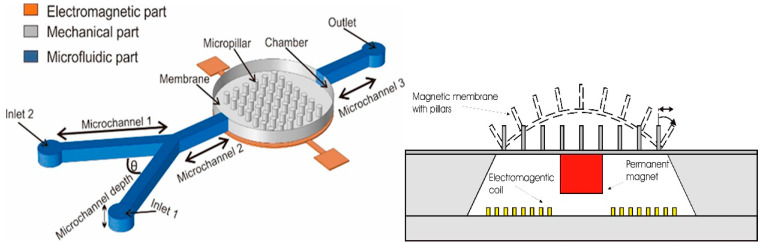
Schematic of a pillar-based active microfluidic mixer (**left**). Polymer pillars deform with the magnetic actuator membrane to induce rotational flow and enhance fluid mixing (**right**) (reprinted from open access) [15].

The manipulation of fluids at the microscale is the focus of microfluidics. Lab-on-a-chip (LOC) systems, also known as MEMS-based microfluidic devices, are utilized in chemical and biological investigations. These gadgets, which enable point-of-care diagnostics, include channels, valves, and micropumps that can precisely control fluid flow [44,45]. When MEMS devices are driven by fluid dynamics for actuation and control, the result is a class of devices known as fluid-drive MEMS devices. These devices are frequently made from pumps, valves, or channels that regulate fluid flow at the nano- or microscale. Polymers like polydimethylsiloxane (PDMS), silicon, or glass are used to produce them. Fluid-drive devices play a crucial role in biomedical applications, especially in lab-on-a-chip systems where the precise control of minute fluid volumes is needed for chemical reactions or diagnostic purposes. Micropumps and microfluidic devices are widely used for drug delivery, environmental sensing, and biomedical diagnostics [44,45,46,47,48,49,50,51]. A magnetic micromixer that uses turbulence to achieve high mixing efficiency is one such system (Figure 6), which comprises embedded polymer pillars in a flexible membrane [15].

#### 2.2.3. RF and Electromagnetic Devices

**Figure 7:**
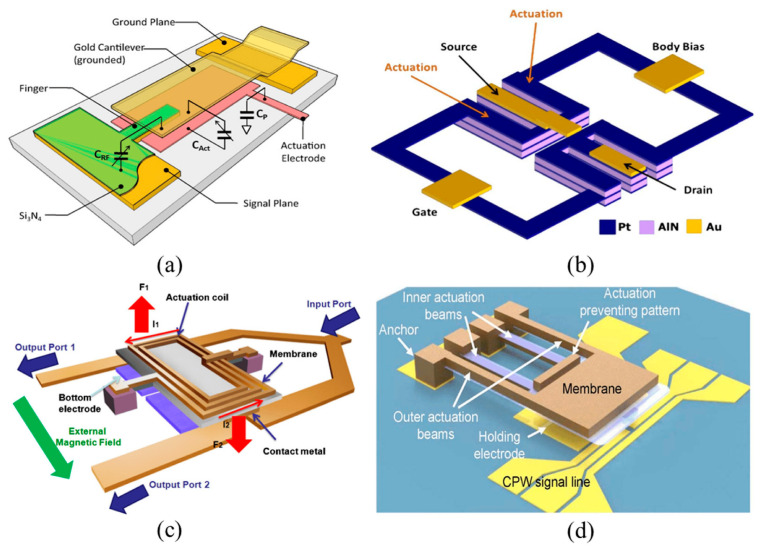
Structures of four RF MEMS switch types based on actuation: (**a**) electrostatic, (**b**) piezoelectric, (**c**) electromagnetic, and (**d**) electrothermal (reprinted from open access) [53].

Wireless communication systems make extensive use of radio frequency (RF) MEMS devices, especially for high-frequency applications. These gadgets have benefits in terms of size, power consumption, and signal integrity, and are applied in the design of cellular handsets, RF switches, adjustable filters, and resonators [52]. RF MEMS devices use a variety of actuation mechanisms, such as electrostatic, piezoelectric, electromagnetic, and electrothermal designs, as shown in Figure 7 [53]. Electromagnetic devices use magnetic fields for both actuation and sensing. Materials with magnetic characteristics, such as cobalt, nickel, and permalloy, an iron and nickel alloy, are used to make these devices. These substances may react with magnetic fields to generate motion or electrical impulses. Transformers, MEMS-based inductors, and micromotors are examples of common devices that make use of electromagnetic principles. MEMS microphones translate sound waves into electrical signals through the use of electromagnetic principles. A relatively new development is the use of nanomagnetic materials to enhance NEMS performance in high-frequency applications [54]. MEMS powered by magnetoelectric (ME) concepts have the potential to transform the internet of things by tackling significant issues related to size, energy efficiency, communication, and environmental adaptation [55].

#### 2.2.4. Energy Harvesters and Thermo-Tensile Devices

**Figure 8:**
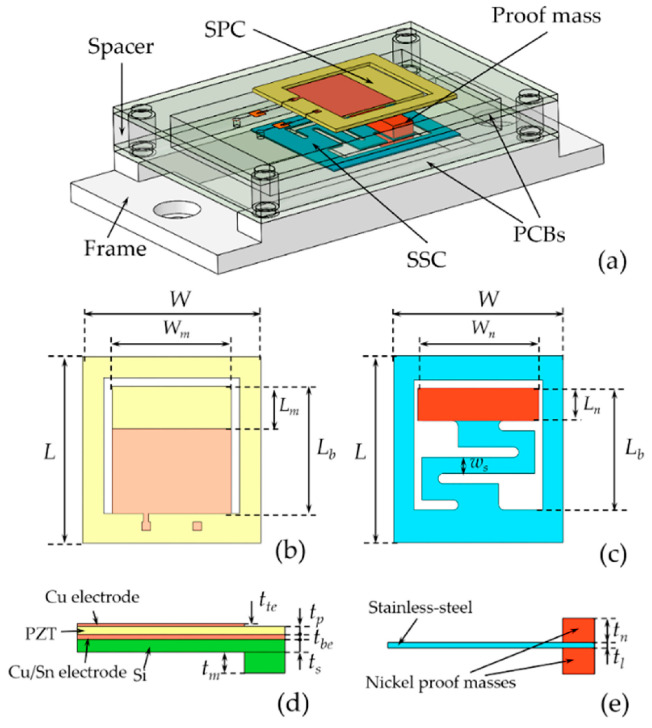
Three-dimensional and cross-sectional schematics of an MEMS piezoelectric energy harvester using stainless steel and PZT cantilevers. (**a**) Three-dimensional schematic of the piezoelectric energy harvesting system. (**b**) Schematic diagrams of the straight piezoelectric cantilever and (**c**) the S-shaped stainless-steel cantilever. (**d**) Sectional views of the piezoelectric cantilever and (**e**) the stainless-steel cantilever (reprinted from open access) [58].

MEMS energy harvesters generate electrical energy from ambient energy sources like temperature gradients and mechanical vibrations. These devices frequently use thermoelectric and piezoelectric materials to energize low-power electronics, such as sensors, in hard-to-reach or remote locations [56,57]. Figure 8 illustrates a typical MEMS energy harvester that uses piezoelectric cantilevers and a frequency up-conversion mechanism [58]. The functioning of thermo-tensile devices is based on the idea of thermal expansion and contraction. Most materials expand when heated, and this characteristic can be used to create mechanical force or displacement. Popular materials like silicon and silicon nitride are preferred due to their favorable mechanical and thermal properties. Metal alloys with high thermal expansion coefficients, such as aluminum or nickel, are also often utilized. Thermal actuators and optical MEMS micro-mirrors commonly use these materials. For optical MEMS, such as digital micromirror devices (DMDs), it is necessary to be able to make large displacements with extremely small temperature changes, which is enabled through the use of actuators with thermo-tensile properties [59].

#### 2.2.5. Optical Devices

MEMS technology, and specifically surface micromachining, has enabled the production of miniature optical devices that have a significant influence on numerous application fields, making them useful for the production, integration, and operation of micro-optical systems [60]. Materials like silicon nitride and silicon dioxide are used to make waveguides, lenses, and mirrors. These materials are employed in optical switches, tunable filters, and displays, as well as in other devices, to regulate light at the micro- and nanoscales [61]. Significant investments in optical MEMS have produced a number of effective components that meet the needs of networks for Lightwave communication [62]. Thermal optical switching, electro-optic switching, acousto-optic switching, and switching based on optical MEMS are among the primary technologies. Depending on the optical technique being used, each optical switching method has distinct performance characteristics. To improve optical switching going forward, it may be necessary to merge several of these technologies together [63].

## 3. Manufacturing Processes

Since the birth of MEMS, the process of manufacturing components for these devices has evolved and advanced. There are diverse manufacturing methods but the most popular include bulk micromachining, surface micromachining, and LIGA. These techniques are essential for molding thin films or wafer materials into high-quality, accurate, and adaptable MEMS devices, such as passive parts, microactuators, and microsensors. The following sections discuss each of these techniques in detail.

### 3.1. Bulk Micromachining

**Figure 9:**
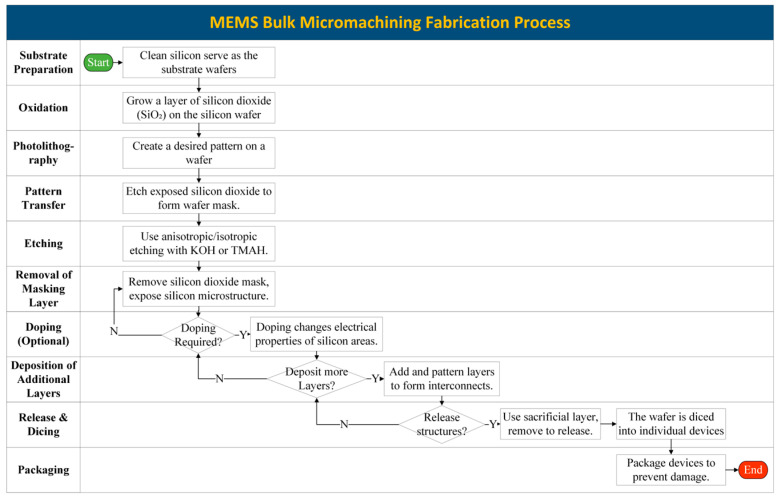
MEMS bulk micromachining manufacturing process.

Bulk micromachining is a fabrication process that involves etching within a substrate, usually silicon because of its crystalline characteristics, to create structures that are useful in the production of MEMS, such as microfluidic channels, cantilevers, and diaphragms. To create accurate and smooth structures inside the substrate, this method employs steps such as substrate preparation, photolithography, etching, cleaning, inspection, and packaging. The steps involved in bulk micromachining are depicted in Figure 9. The following sections discuss these steps in greater detail.

#### 3.1.1. Substrate Preparation

The proper preparation of the substrate when manufacturing MEMS makes it possible to successfully produce these devices. Choosing the right wafer, cleaning it, generating a silicon dioxide layer, applying photoresist, transferring a pattern using photolithography, developing the photoresist, etching away undesirable material, and checking for flaws are all steps in the preparation of MEMS substrates. Various materials have been studied for attachment to the die and multiple sample preparation techniques have been examined to determine their effects on measurement outcomes [64]. Smooth, clean surfaces are necessary for these operations in order to mix the layers. To achieve optimal adhesion, minimize flaws, and guarantee the precision, reliability, and performance of the device, proper substrate preparation—which includes cleaning, applying photoresist, pattern transfer, and making sure the surface is smooth—is essential in MEMS manufacturing. Another means of preparing substrates is, after cleaning silicon substrates in a piranha solution, using the microfabrication technique to create an isolation-enhancing thermal oxide layer. On oxidized wafers, photolithography, etching, and deposition produce exact patterns and layers, while doping modifies electrical characteristics via ion implantation. Sacrificial layer removal releases moveable elements, while lift-off eliminates undesired materials. MEMS devices are completed for testing and use via bonding, packing, sealing, and integrating the microstructures. To create multi-layered MEMS structures, these procedures can be repeated using other materials and masks [65].

#### 3.1.2. Photolithography

Photolithography, which uses light to imprint a design from a mask onto a substrate composed of photosensitive material, is a crucial stage in the production of MEMS. Accurate and reproducible patterning is required to produce complex microstructures in these MEMS devices. A photomask, typically a glass plate or plastic film covered with a non-UV-transparent film, is prepared for photolithography and placed on a silicon wafer coated with photoresist. This exposes or protects regions against UV light, creating a pattern on the wafer. When exposed to UV light, the positive photoresist dissolves in the developer, revealing the pattern on the mask. In contrast, exposed regions of negative photoresist become cross-linked and intractable. While negative photoresist is more durable and chemically resistant, making it suitable for structural or protective layers, positive photoresist excels at producing high-resolution, intricate patterns, making it perfect for MEMS manufacturing. The design and process requirements determine the decision. The desired design is formed by UV light exposure through the open portions of the mask, and a master mold is created when the remaining photoresist is removed [66].

**Figure 10:**
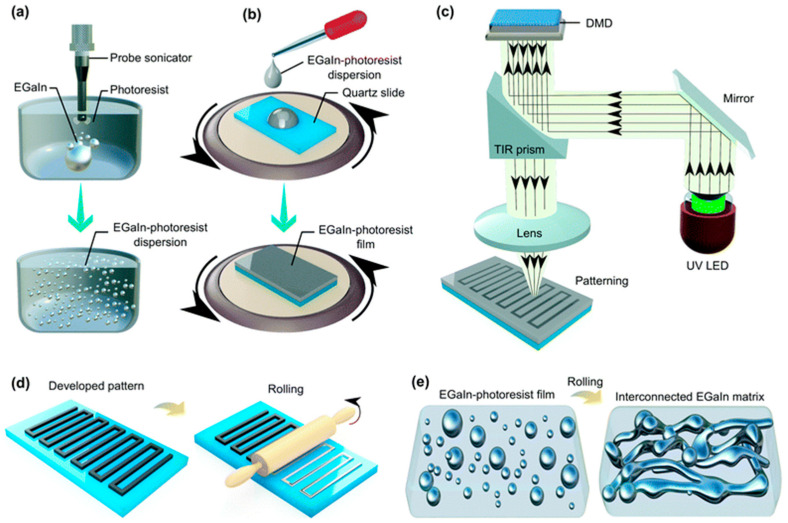
The photolithography process in microfabrication using liquid metal (**a**–**e**) (reprinted with permission from RSC) [67].

Soft lithography uses elastomeric stamps, molds, or masks to create micro- and nanoscale patterns, offering more flexibility than traditional photolithography. It is particularly suited for microfluidic devices and flexible or biological MEMS components. Polydimethylsiloxane (PDMS) is often used to create stamps that transfer patterns onto substrates. Figure 10 illustrates the photolithography process used in microfabrication, which enables the precise patterning of liquid metal patterns at the micro- and nanoscales.

#### 3.1.3. Wafer Bonding

Bonding is a process used to join two or more silicon wafers or other materials to create a single MEMS device. In MEMS devices, adequate bonding guarantees structural integrity, functioning, and dependability. While strong, consistent bonding preserves the layer alignment, which is essential for the accurate operation of components like sensors, actuators, and microfluidic channels, poor bonding increases the danger of delamination, leakage, or failure under stress.

Common bonding techniques include the following: (1) anodic bonding, which uses an electric field at high temperatures to create strong, hermetic seals, (2) fusion bonding, which fuses ultra-clean surfaces at high temperatures for excellent mechanical stability, and (3) adhesive bonding, which uses adhesives at lower temperatures. Depending on the unique requirements of the MEMS device, each strategy offers unique advantages. For high density and low parasitic impedance, flip-chip bonding can also be used to join ICs face-down to substrates or other ICs with solder bumps.

By using methods including direct bonding, anodic bonding, interlayer bonding, and wafer bonding, also known as plate-joining microprocessing, several micro-level layers are created. Barajas-Valdes explored the nanomechanical characteristics of pure aluminum and aluminum–boron thin films created via magnetron sputtering, including adhesion [68]. Techniques including wafer bonding, anodic bonding, interlayer bonding, and direct bonding are used to produce a number of micro-level layers. Barajas-Valdes investigated pure aluminum and aluminum–boron thin films produced via magnetron sputtering, finding that the addition of boron greatly improved the mechanical qualities. Compared to pure aluminum, aluminum–boron films demonstrated superior adhesion and hardness, making them more resilient and appropriate for MEMS and NEMS applications.

**Figure 11:**
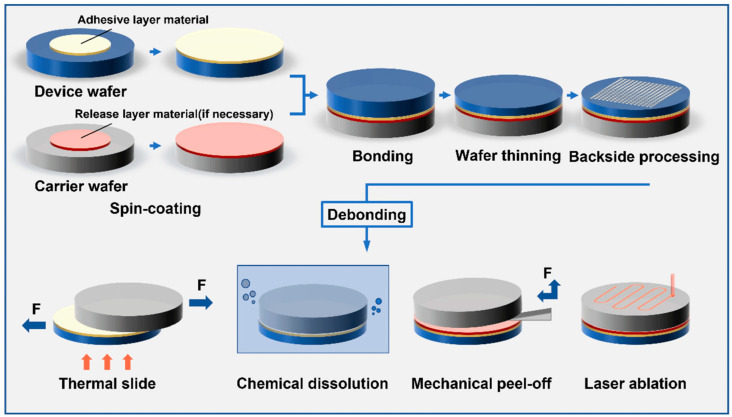
Overview of the temporary bonding and debonding process flow, where F denotes a possible external force applied to facilitate the debonding stage (reprinted from open access) [71].

The material target, substrate type, and sputtering circumstances all affect the mechanical behavior of the films that are deposited on silicon wafers and glass substrates. Compared to pure aluminum films, composite films demonstrate higher adherence. The effects of these composite thin films on adhesion forces and surface roughness were examined using an AFM microscope in conjunction with the Rabinovich and Rumpf models. Together, the Rabinovich and Rumpf models provide thorough knowledge of adhesion by taking into consideration material characteristics and nanoscale surface roughness, allowing for more precise predictions of thin film behavior and surface interactions [69]. The outcomes were compared to those of single Ag and Au layers. In comparison to single-layer Ag and Au films, the composite Ag-Au thin films demonstrated improved adhesion forces and decreased surface roughness. According to the adhesion models and AFM research, the synergistic interaction between silver and gold results in improved substrate bonding and a consistent film shape, which in turn improves mechanical stability and film quality [70]. A crucial stage in the manufacturing of semiconductors, temporary bonding offers durability throughout back-end processing and wafer thinning. As seen in Figure 11, quality checks are conducted after an adhesive is used to fuse a carrier wafer to the device wafer. Debonding employs either a single adhesive or an additional release layer, depending on the technique—thermal sliding, chemical dissolution, mechanical peel-off, or laser ablation. To improve sustainability and cut costs, the carrier wafer is cleaned for reuse after processing, and the device wafer is separated.

#### 3.1.4. Etching

The technique of bulk micromachining requires selectively etching structures within a silicon substrate in order to develop MEMS. Etching is the process of creating desired features in silicon: wet and dry etching techniques are the two primary categories. The choice of etching type in micromachining technology is contingent on availability and need. Wet anisotropic etching is commonly chosen over dry etching because it reduces fabrication costs, which is essential to industrial success. This technique typically uses alkaline solutions like potassium hydroxide (KOH) or tetramethylammonium hydroxide (TMAH) to produce precise and smooth shapes inside the substrate. All other crystal planes etch quickly in the wet anisotropic etchants; however, the {111} Si planes etch relatively slowly. Therefore, only squares and rectangles can be produced with great precision in {100} silicon wafers. One well-known feature of wet anisotropic etchants is the undercutting at mask edges that are not aligned along the <110> directions on the {100} Si surface [72,73]. On the {100} silicon surface, mask edges are aligned in the <110> directions to avoid undercutting during MEMS manufacture. Crystallographic planes are etched at varying rates by wet anisotropic etchants such as KOH and TMAH; for accurate sidewalls, <111> planes form natural stop points for the etching. While misalignment jeopardizes dimensional correctness, structural integrity, and dependable device performance, proper alignment guarantees accurate microstructures. The functionality and consistency of MEMS and integrated circuits depend heavily on precision.

Deep reactive ion etching (DRIE) using SF_6_/O_2_-based high-density plasmas at cryogenic temperatures is another method for creating MEMS structures using silicon [74]. For the production of MEMS, DRIE employing SF_6_/O_2_-based high-density plasmas at cryogenic temperatures is widely regarded as preferable, especially when it comes to producing deep, extremely accurate, and high-aspect-ratio structures. Even though wet and dry etching methods are easier and less expensive, they usually do not work as well for precisely generating intricate, deep designs. Therefore, although DRIE is more expensive and requires more specialized equipment, it is frequently superior to wet/dry etching for MEMS manufacturing.

#### 3.1.5. Release, Assembly, and Packaging

In order to liberate microstructures from their substrate, sacrificial layers must be removed during the release process in MEMS production. Wet etching (such as hydrofluoric acid), vapor-phase etching (to lessen stiction), and dry etching (such as plasma etching for accuracy and contamination control) are common techniques. Maintaining the structural soundness, functioning, and dependability of MEMS devices requires this phase. In MEMS manufacturing, the assembly process combines MEMS devices with electrical circuits or incorporates microfabricated components. Functionality and limitations are taken into consideration when selecting techniques like flip-chip bonding, wire bonding, and adhesive bonding. Microsensors, microactuators, and integrated microsystems are examples of fragile components whose performance and functioning are guaranteed by precise robotic manipulation and automatic alignment. Similarly to solid-state relays, MEMS can be manufactured in batches, enabling the simultaneous creation of several devices. This lowers expenses while helping to maintain constant quality across all devices. The production of MEMS in large quantities reduces costs and supports their broad use in a variety of applications [75]. It is essential to prevent mechanical damage and improve packaging seals to prevent contamination and stop switch contact degradation. There are currently no standardized criteria for evaluating dependability, such as lifespan and mechanical characteristics, and the causes of failure are not well understood. Furthermore, mass production is restricted by the high cost of packaging, which frequently surpasses the manufacturing costs [76].

### 3.2. Surface Micromachining

**Figure 12:**
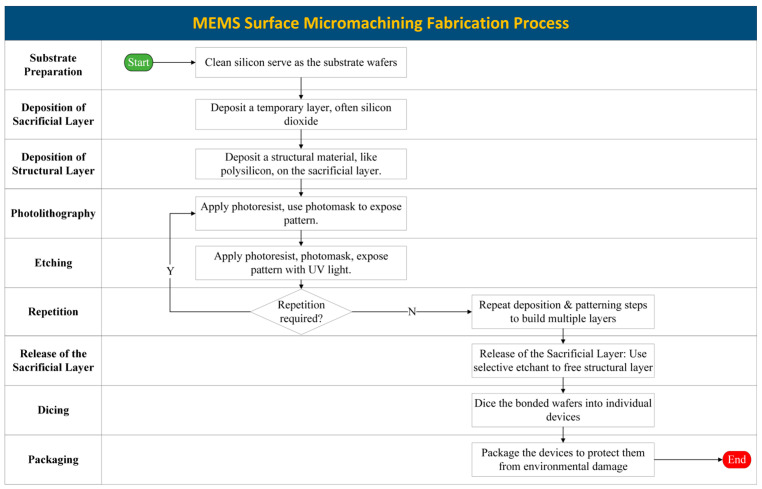
MEMS surface micromachining manufacturing process.

There are many similarities between bulk micromachining and surface micromachining. Surface micromachining is an MEMS production process that uses etching and thin-film deposition to create structures on a substrate’s surface. In this procedure, a sacrificial layer is deposited and patterned, and then a structural layer is added. The freestanding MEMS structure remains when the sacrificial layer is removed. Figure 12 presents the surface micromachining process. Microsensors, microactuators, and other intricate microsystems are frequently developed using this technique. Surface micromachining reduces costs by facilitating batch processing and enables the integration of layers to improve device performance. However, issues like residual stress and the requirements for exact alignment still need to be worked out. Surface micromachining is still a crucial technique for creating complex microsystems with enormous potential in a variety of high-tech industries, despite these difficulties.

For example, one device that relies on this technique is a magnetic actuator with a maximum displacement of 50 µm that was created by embedding magnetic NdFeB microparticles in a nylon 12 matrix. Fogel et al. developed fully metallic electrothermal actuators that deflect when a current pulse is supplied using laser-induced forward transfer (LIFT) technology [77]. There were multiple steps in the fabrication process. After creating a polyimide (PI) sacrificial layer, AZ4620 photoresist was spin-coated to create anchor through-holes. After the PI layer was removed via etching, AZ4620 was employed again as a mask to define the actuator structure. The top electrode was made by sputtering a thin layer of aluminum that was patterned using O_2_ plasma and phosphoric acid etching. The free-standing actuator structure was left behind after the PI sacrificial layer was removed in an oxygen plasma atmosphere. The electrothermal effect caused this actuator to deflect, which makes it appropriate for a range of MEMS applications [78].

Additionally, a hybrid surface/bulk micromachining (SBM) process model was developed, enabling the creation of an MEMS using bulk silicon. Starting with a silicon wafer, structural patterns are defined using reactive ion etching, followed by passivation with an oxide film and further etching to set the sacrificial gap dimensions. The final release is achieved via undercutting with aqueous alkaline etchants, resulting in clean surfaces. The SBM process enables the fabrication of single-crystal silicon structures with various thicknesses and sacrificial gaps. This method offers a viable alternative to existing micromachining techniques, leveraging the benefits of single-crystal silicon [79].

### 3.3. LIGA

In order to construct complicated MEMS devices from materials such as metals, ceramics, and plastic that cannot be produced using conventional silicon processing methods, LIGA technology (Lithography, Galvanoformung, Abformung) can be utilized [65]. The microfabrication technique known as LIGA, or Lithography, Electroplating, and Molding, produces high-resolution, high-aspect-ratio microstructures. Molding is used to shape materials, electroplating is used to create metal structures, and deep X-ray or UV lithography is used to design patterns. LIGA is perfect for creating precise metal, polymer, and ceramic components that are frequently utilized in high-performance MEMS such as precision gears, microturbines, and micro-optics.

After defining patterns in a thick layer of photoresist using deep X-ray lithography, electroplating is used to create long-lasting metal structures like copper, nickel, or gold. Complex microstructures can be produced by using these metal structures as molds to duplicate parts in metals, polymers, or ceramics. High precision, material adaptability, and scalability are among the benefits for both large-scale production and prototyping. However, this method also has drawbacks, like the expensive and complicated equipment required, which makes it less appropriate for low-volume manufacturing. All things considered, LIGA is an effective technique for producing long-lasting, highly precise microcomponents, especially in sectors like microelectronics, medical devices, and aerospace.

Table 1 illustrates the three key micromachining methods mentioned in previous sections: (1) bulk micromachining, which creates deep, robust 3D structures for sensors and cavities, (2) surface micromachining, which forms thin, precise surface microstructures using sacrificial layers, ideal for MEMS with moving parts, and (3) LIGA, which produces high-aspect-ratio, detailed microstructures in metals or polymers for applications like microgears and molds, with differences in depth, precision, cost, and strength.

## 4. Biomedical Applications

Devices such as sensors, actuators, optical switches, resonators, chemical and thermal sensors, electrically driven devices, medical equipment, microfluidic devices, and optoelectronic devices are all included in the broad category of MEMS. For biomedical applications, many of these devices are implemented while also being frequently integrated within or around the human body. Every device has a distinct function, and the following sections will discuss some of the prevalent applications of MEMS in biomedical settings.

### 4.1. Biosensors

**Figure 13:**
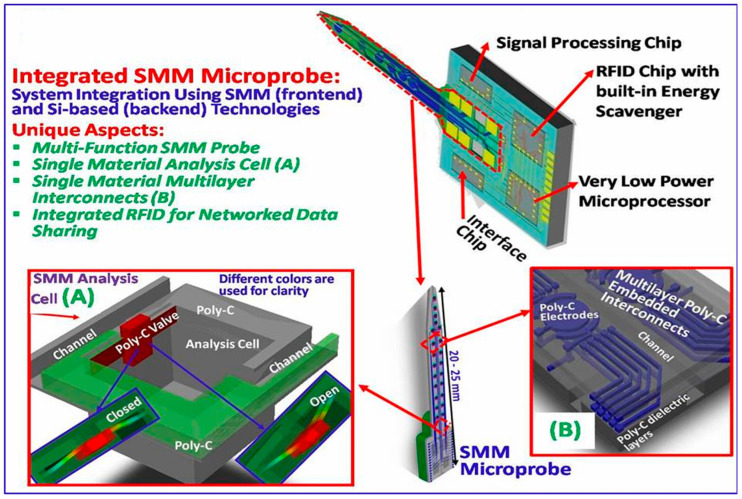
(**A**) Nanoscale electromagnetic analysis of biological samples by the SMM analysis cell, (**B**) Integration of SMM microprobes with biosensing systems for real-time biological detection (reprinted with permission from open access) [88].

MEMS biosensors are widely used for point-of-care applications in a wide range of industries, including drug research, environmental monitoring, food safety, and healthcare [83]. For the development of integrated, scalable, and sustainable bio/chemical sensing systems used in environmental monitoring, disease diagnosis, drug discovery, and food quality assessments, MEMS biosensors are essential [84,85]. MEMS biosensors operate at the microscale, have high sensitivity, accuracy, and precision, and can easily be mass-manufactured, allowing for new developments in a wide range of biomedical applications. In addition to being dependable, they are lightweight, energy-efficient, and appropriate for challenging conditions. MEMS biosensors are employed in the tracking of fitness and medications, wearable technology, and medical diagnostics [86]. These biosensors can also assess toxins in the environment and food, identify diseases, and monitor either chemical or physical signals and biological systems [87]. The ability of biosensors to collect data quickly, accurately, and in real time across a variety of domains makes them indispensable. Figure 13 shows a single-material MEMS (SMM) system with multi-functional probes that can record individual neuron activity and detect extracellular neurotransmitters.

#### 4.1.1. Pressure Sensors

MEMS pressure sensors are widely used in the medical field to track pressure in a given environment. These pressure sensors are classified according to their operating principles, such as their use of (1) differential versus partial pressure, (2) high versus low pressure, or (3) a sensing component, including piezoelectric, piezoresistive, and capacitive components [89]. For the diagnosis and treatment of many biological disorders, pressure sensing is essential. A biomedical pressure sensor must be designed, simulated, and optimized. This includes choosing the best materials and structures, modeling behavior with simulation tools, optimizing the design for improved performance, testing under real-world settings, and integrating the sensor into a medical device. This procedure helps guarantee precise, trustworthy pressure monitoring for the treatment of medical disorders [90].

MEMS pressure sensors, particularly the capacitance and piezoresistive types, are renowned for their exceptional sensitivity and accuracy [91]. For example, a semiconductor-based MEMS pressure transmitter with a central vacuum chamber coated in an oxide with a silicon base and a thin silicon membrane was created and its ability to precisely measure blood pressure was examined [92]. These devices were shown to be able to assess blood flow in the wrist’s radial arteries, which reveals concentration shifts and membrane displacements that are used to determine an adult’s heart rate [93]. A commercial interface micromachined piezoresistive pressure device that uses a polysilicon method can also measure arterial blood pressure with an accuracy of more than 2 mmHg and is used in balloon angioplasty [94]. Meena also emphasized the development, uses, and significance of pressure sensing in the diagnosis and treatment of biological disorders [95].

MEMS capacitive pressure sensors are ideal for many industrial, consumer electronics, medical, and automotive applications because of their high sensitivity, low power consumption, compact size, high accuracy, wide pressure range, robustness, long-term stability, and cost. Precision microsurgery, targeted therapies, controlled drug distribution, and disease detection and diagnostics are just a few of the biomedical applications in which this technology shines. By enhancing early detection, accurate diagnosis, efficient treatment, and minimally invasive surgical procedures, its adaptability improves healthcare and improves patient outcomes [96]. Since capacitive pressure sensors use a silicon membrane as the movable electrode and a gold metal layer on a glass substrate as the fixed electrode, they are well known for having superior long-term stability and better reliability under temperature variations compared to piezoresistive MEMS sensors [97]. Capacitive pressure sensors have also been shown to facilitate the development of sustainable, scalable, and integrated bio/chemical sensing systems for environmental monitoring, disease detection, food quality evaluations, and drug discovery [34]. One such device has a higher sensitivity for liquid samples than cantilever-based biosensors because of its architecture, which improves the noise-to-signal ratio [98].

The need to manage parasitic capacitance, aging effects, humidity sensitivity, mechanical stress during packing, electrostatic discharge protection, thermal drift, advanced signal processing requirements, and the high cost of high-quality components are a few challenges that face MEMS capacitive pressure sensors. The need for accurate temperature corrections, as well as the need to reduce noise and signal interference, manage mechanical fatigue, create appropriate packaging, attain dependable calibration, increase dynamic range, and avoid biofouling, are some of the difficulties that biomedical MEMS pressure sensors as a whole must overcome. Innovative design, cutting-edge materials, and ongoing advancements are needed to resolve these problems.

#### 4.1.2. Novel Applications

Complex fabrication, material selection, environmental sensitivity, integration issues, power management, calibration accuracy, scalability, reliability, data security, mechanical fatigue, biocompatibility, signal processing, thermal management, interdisciplinary collaboration, economic viability, and regulatory approval are some of the particular difficulties that novel MEMS approaches must overcome. To overcome these issues, advanced engineering and constant innovation are needed. Retaining sensitivity and accuracy when downsizing sensors for new applications may be challenging. Harsh or variable environmental conditions, such as corrosive environments or excessively high or low temperatures, can affect the lifespan and functioning of sensors. Modern systems and technologies present difficulties with integration, making compatibility with other sensors and the creation of a seamless digital system interface challenging. As new applications often require lower power consumption, these sensors’ capabilities are pushed. Another significant challenge is reducing production costs without sacrificing superior accuracy and dependability [99].

The innovative application of rough-surfaced polyvinylidene fluoride nanofibers in MEMS solves some of these issues by significantly boosting viral sensitivity while affecting electrical resistance and displacement [100]. Another novel small fiber-optic sensor has been shown to assess the arterial pressure of a Yorkshire pig model. Blood pressure readings in the opposing coronary arteries and the aortic arch have also been used for tracking, using standard invasive manometry as the reference [101]. By successfully recording blood pressure data in the right coronary artery and aortic arch and by tracking the pressure decrease caused by an inflated balloon that mimicked stenosis, the sensor showed potential for fractional flow reserve applications [102]. MEMS hydrodynamic biological sensors also show promise in applications where increased mobility and stability in biological systems is necessary, despite having problems with automation and downsizing [103].

**Figure 14:**
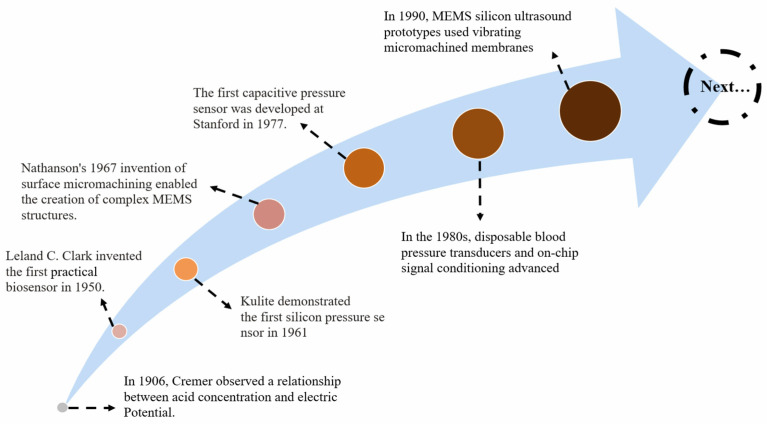
MEMS biosensor history [91,104,105,106,107,108,109,110].

Over the past century, MEMS biosensors have seen remarkable development. Cremer’s discovery of the connection between acid content and electric potential in 1906 marked the beginning of this movement. Using this success as a building block, Leland C. Clark created the first practical biosensor in 1950. The creation of MEMS silicon ultrasonic prototypes with vibrating micromachined membranes in 1990 was one of many subsequent developments. Figure 14 provides a comprehensive roadmap of this discovery.

### 4.2. Wearable Devices

Because of their small size, low power consumption, precise and real-time health and activity monitoring, high sensitivity, adaptability, ease of integration, affordability, convenience, and durability, MEMS wearables are crucial in both fitness and healthcare industries. Recently, flexible wristbands based on MEMS components have been developed in response to the increased demand for affordable, user-friendly wearable technology for ongoing cardiovascular health monitoring [111]. Advances in manufacturing techniques and material science have significantly increased the profile of sensors’ use in health information technology, even if only a small number of devices that use sensors for real-time cardiovascular monitoring are clinically validated [112]. Through the non-intrusive collection of real-time physiological indications utilizing a range of sensors, MEMS are also integrated into fashionable devices to enhance clinical diagnosis for consumers.

The need for power management, additional miniaturization and integration, user comfort and ergonomics, durability, reductions in signal interference, the preservation of calibration and accuracy, data privacy and security, manufacturing consistency, and regulatory compliance, as well as cost limitations, are some of the difficulties faced by MEMS wearables. Continuous innovation and further knowledge of user requirements and standards are necessary to address these issues.

### 4.3. Transducers

MEMS transducers combine both mechanical and electrical components to transform energy forms at the microscale. Pressure sensors, accelerometers, gyroscopes, and microphones are a few examples that provide accurate and dependable measurements and control in a variety of sectors. Bio-transducers, devices that convert biological impulses into observable outputs, are essential components of many biomedical MEMS applications, especially when combined with bioreceptors and microelectronics [113].

**Figure 15:**
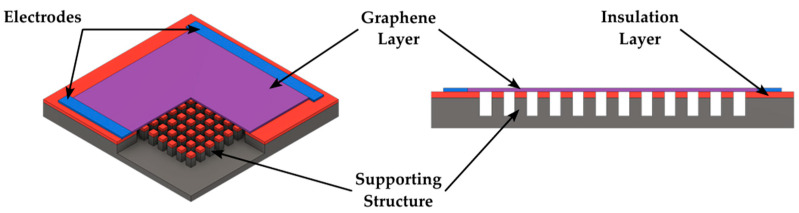
Three-dimensional schematic and cross-sectional view of a thermoacoustic speaker (reprinted with permission from open access) [116].

Microcantilevers can also be used as sensors and transducers. MEMS biosensors using microcantilevers are excellent at detecting antibody–antigen binding, which is essential for diagnosing HBV and H1N1, according to COMSOL Multiphysics [114]. Assessed using COMSOL models and meticulous fabrication techniques, an implantable, real-time molecular detector for blood analysis utilizing MEMS technology exhibited considerable promise for prompt diagnosis during disease outbreaks. Lakshmi and his colleagues designed and simulated several microcantilever configurations using COMSOL Multiphysics simulation software in order to identify the best design for integration with an electro-osmotic pressure sensor for accurate glucose level measurements. The mechanical behavior, sensitivity, and non-linear features of each configuration were then investigated using finite element simulation [115]. Figure 15 depicts a 3D schematic and cross-sectional view of a thermoacoustic speaker, showing how the expansion and contraction of a heated material produces acoustic vibrations, which in turn transform thermal energy into sound waves. Like other MEMS devices, MEMS transducers have difficulties with cost, scalability, environmental sensitivity, power consumption optimization, material selection, fabrication complexity, and electronics integration. In order to improve methods, materials, and overall device performance, these problems require ongoing research and development.

### 4.4. Microvalves

MEMS microvalves are essential to chemical processing, biomedical engineering, and lab-on-a-chip technologies because they can handle small fluid volumes that conventional devices cannot. The optical clarity, biocompatibility, mechanical rigidity, and thermal stability of glass make it an ideal material for microscale devices such as microvalves because it combines strength, stability, safety, and clarity to satisfy the exacting needs of cutting-edge technological applications [117]. One of the well-known applications of lab-on-a-chip is in high-stroke, reasonably priced electromechanical benders, where it takes the place of conventional solenoid-based microvalves in water heaters to regulate gas flow. To determine the ideal elastic deformation behavior for a light-responsive microvalve, Cugno compared two production techniques [118]. The CNT-PDMS combination’s good light response was verified through testing and the optimal design was determined. This invention improves precision and coupling possibilities in sophisticated systems by enabling the precise control of liquids in applications like medical instruments, lab-on-a-chip systems, and smart materials.

To create negative pressure, Richter et al. are creating multi-stage, high-performance micropumps [119]. Up to 82.1 kPa of negative pressure was attained through using a cascade of three micropumps. This development is important for applications including the processing of chemicals and biomedical equipment, particularly microfluidic assemblies that need accurate low-pressure conditions. These microvalves improve safety and efficiency in the chemical manufacturing process by providing exact control over reactions and reagent mixing. Key MEMS components like micropumps and components ranging from basic valves to intricate biomimetic devices can be developed through the use of magnetic polymer combination sheets [120]. Critical parts, including ion sources, mass analyzers, and detectors, have been successfully microfabricated using MEMS technology. By offering accurate, effective, and compact approaches, researchers have transformed a number of academic and technological domains, including analytical chemistry and biomedicine [121]. In biomedical applications, MEMS microvalves enable lab-on-a-chip devices that miniaturize the laboratory processes, enhancing the efficiency and portability of diagnostics and drug delivery. This has applications in biology, medicine, and environmental preservation [122]. Because they handle biological materials more gently and cause less clogging, valveless micropumps are preferred. Their valve-free design improves fluid flow and reduces particle trapping, both of which are critical for fragile biological samples. These pumps are perfect for delicate and precise uses in current technology because they are adaptable, have a lower flow resistance, and are easier to build and maintain [123]. MEMS can also be used in high-tech, energy, medical, and environmental applications. In order to create MEMS components with characteristics that are suited for a variety of applications, such as micropumps and valves, magnetic polymer composite films are essential. Microvalves, micropumps, micromixers, microsensors, drug delivery systems, and magnetic labeling and separation systems are examples of recent developments in magnetic films made from polymers and MEMS [120].

### 4.5. Actuators and Circuits

MEMS technology offers accurate, effective, and adaptable solutions in a variety of fields. Wearable sensors require additional clinical testing despite advancements. Using developments in microfabrication technology, a generalized MEMS combines electronics, sensors, actuators, and mechanical parts. Smaller, quicker, and more effective devices can be produced thanks to MEMS technology, especially in optical and radio frequency systems. Sensors, displays, and fiber-optic switches are examples of optical MEMS applications [124]. One research effort discusses MEMS sensors, actuators, the phenomena they may identify and respond to, and the main obstacles facing the sector [125]. By producing pulsed and quasi-steady jets, these types of actuators successfully reduce cavity oscillations. The goal of flow control is to alter a flow’s inherent state to produce a more advantageous situation, like a decrease in noise or drag [126]. An on-chip micro-syringe pump has also been shown to overcome the difficulties of downsizing conventional syringe pumps by using electro-conjugate fluid (ECF) rather than piston technology and electrohydrodynamic (EHD) flow [121].

Another work examines and classifies a broad variety of electrostatic actuators created in the previous five years, encompassing distinct designs and their uses [127]. High displacements in the X, Y, and out-of-plane (Z) directions are provided by this integration, guaranteeing excellent alignment and exact control. Another platform, which represents a major leap in MEMS technology, is ideal for applications that demand minute modifications and high precision [128]. As a sensitive plant, Mimosa pudica, for instance, manages the opening and closing of its valve in response to outside factors, much like a microvalve system. This inherent process motivates the creation of MEMS microvalve devices that recognize inputs and modify themselves in response, providing accurate fluid control in a range of applications, as well as improving responsiveness and flexibility [129].

In biomedical applications, actuators and circuits are crucial because they allow for precise control and automation in equipment such as implants, surgical instruments, prosthesis, and diagnostic systems. Circuits process signals, regulate timing, and handle feedback, whereas actuators carry out physical activities like movement or medication delivery. They work together to power cutting-edge technology that significantly increases the efficacy and personalization of medical care, such as brain–machine interfaces, smart implants, robotic limbs, and rehabilitation equipment.

## 5. Challenges and Prospective Trends

### 5.1. Current Challenges and Potential Solutions

#### 5.1.1. Biocompatibility, Safety, and Durability

Biocompatible materials do not harm living tissue in the short or long term. Ensuring biocompatibility helps to prevent adverse effects, such as inflammation, immune responses, or long-term toxicity. For example, in the application of glucose monitoring for managing diabetes, a device has been proposed that will continuously measure glucose levels in subcutaneous tissues for an extended period of time [130]. There is an inverse relationship between biodegradability and durability. Depending on the application, one may be preferred over the other, leading to the challenge of balancing the lifetime of each device. For devices designed to collect data over long-term periods of time, it is advantageous to build from biocompatible materials that do not degrade over the lifespan of the product. On the other hand, if disposable MEMS devices are required in the application, it is critical to select the appropriate biodegradable material. If the material is not chosen carefully, the device could irritate or even damage the tissue, potentially causing the sensor readings to be incorrect as well as harming the individual. The materials used most commonly in MEMS devices can be arranged into three groups: silicon-based materials, metals, and polymers [131].

Materials such as silicon, silicon dioxide, gold, silicon nitride, titanium, silicon carbide, and SU-8^TM^ show biocompatibility, with silicon nitride, gold, silicon dioxide, and SU-8^TM^ additionally preventing biofouling [132,133,134]. SU-8^TM^ was investigated as a silicon wafer photoresist because of its biocompatibility; however, issues with mechanical delamination exclude this application until further research is conducted to find better bonding methods [133]. While some of these materials may be biocompatible, another concern is the ability to use them in the manufacturing of MEMS, which have a very different fabrication process to that of traditional implants [134].

Advancements in materials science are crucial to the progression of MEMS manufacturing, especially in the biomedical field due to the need for biocompatible and durable devices [3]. Currently, monocrystalline silicon is the most prevalent material used in manufacturing MEMS, being commonly used for both the substrate and structural materials [135]. Many materials are biocompatible but eventually suffer as they are biodegradable as well [136].

Hydrogel-based actuators show promise in many applications, such as sensors, optical devices, microfluidic devices, walkers and swimmers, 3D microfabrication, and stimuli-responsive surfaces [137]. The response time for these actuators actually improves when the scale decreases [137]. One caveat of this technology is that it must reside within an aqueous environment for the best results. However, in biomedical applications, this does not present much of a challenge because of the aqueous nature of the human body.

**Figure 16:**
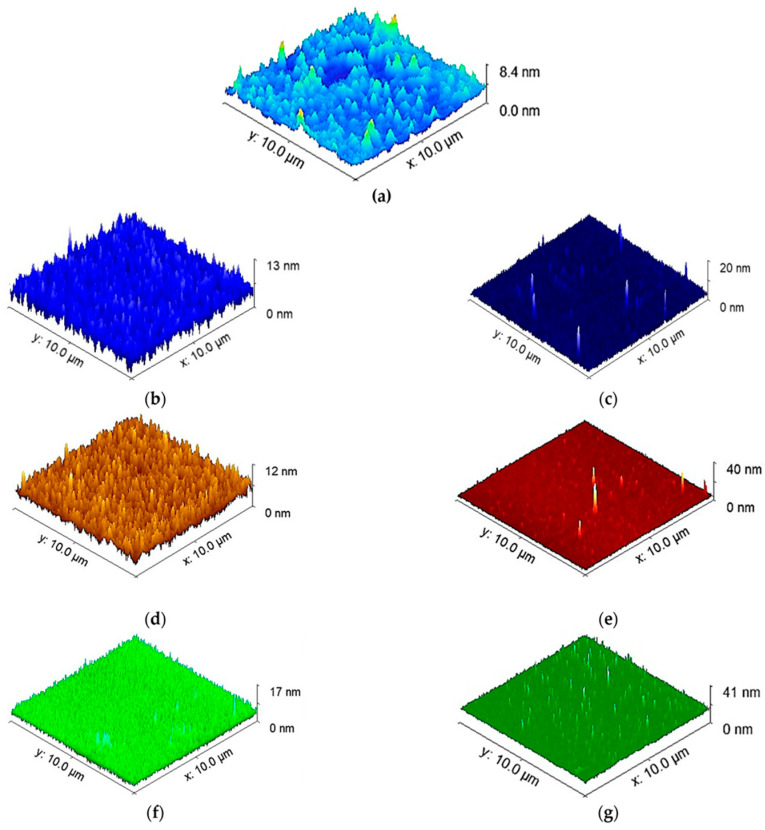
Three-dimensional topographies for (**a**) uncoated silicon substrate; (**b**) 120 nm Ag coating thickness; (**c**) 500 nm Ag coating thickness; (**d**) 120 nm Au coating thickness; (**e**) 500 nm Au coating thickness; (**f**) 120 nm Ag-Au compound coating thickness; and (**g**) 5000 nm Ag-Au compound coating thickness (reprinted with permission from open access) [70].

Factors such as surface topography, roughness, and chemistry also play a role in the adhesion of cells to the MEMS device for tissue engineering applications [131]. Surface topography and roughness both have a direct effect on the surface area available for cell attachment, while surface chemistry impacts the level of cell adhesion and protein adsorption [131]. Figure 16 illustrates the impact that factors such as the coating material and thickness have on the topography of an object, which plays a large role in the adhesion force experienced at the object surface [70]. Better adhesion promotes longer-lasting and more resilient devices, especially in applications within the human body.

**Figure 17:**
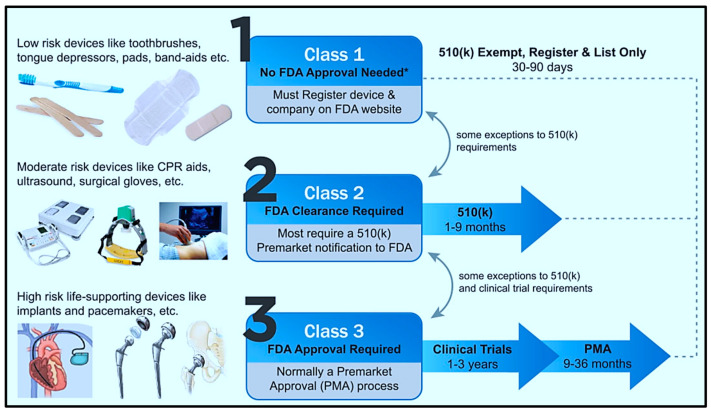
Medical device classes according to the U.S. FDA [138].

When introducing new technologies within the medical field, especially ones that are meant to be placed directly within the human body, certain regulations must be met to ensure individual safety. Figure 17 provides an overview of the classifications of medical devices produced in the U.S., and the related FDA approval required. This can present a barrier to the development of novel biomedical MEMS technologies due to the extensive approval process that must be handled beforehand.

#### 5.1.2. Energy Management

Commercial success often comes from having the most advanced technology in a given field. One technological challenge that still remains in biomedical MEMS devices is the need to provide power to these devices in the final environment. Powering nanoscale actuators is difficult, because batteries are not yet nanoscale. Any devices used to power the MEMS devices deployed in biological environments will need to be biocompatible, efficient, and reliable [4]. One solution could potentially come in the form of in situ energy harvesting, where environmental stimuli are transduced into electrical energy [4].

Triboelectric nanogenerators have been gaining more attention in the last decade. These devices rely on the friction from two surfaces sliding against each other to generate electricity. They have only recently been successfully applied to the field of MEMS. Two examples of novel self-powered devices built using triboelectric nanogenerators are an MEMS vibration sensor and an MEMS accelerometer [139,140]. However, this technology is rarely implemented in biomedical applications at the micro- and nanoscale.

#### 5.1.3. Manufacturing and Scalability

**Figure 18:**
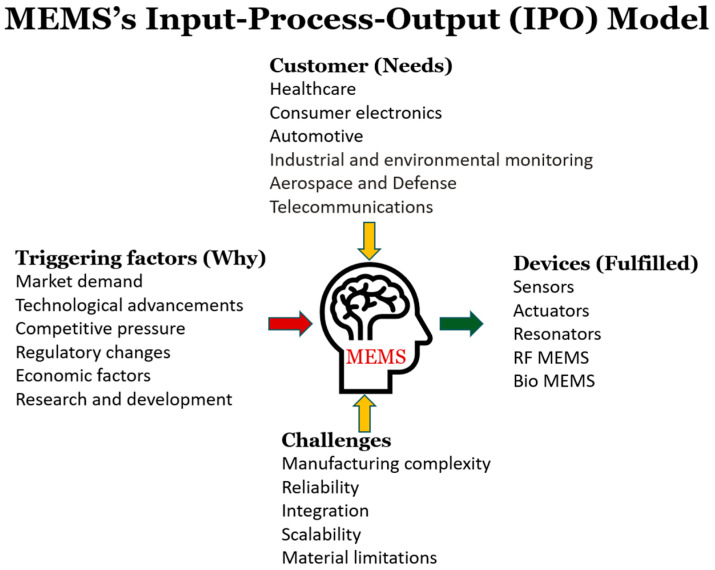
MEMS IPO model.

Post-processing increases the production time and the associated costs due to the delays and equipment needed. Monolithic manufacturing is a dominant fabrication strategy in the creation of MEMS devices. When this fabrication method is implemented with CMOS, post-processing is required, which increases costs [136]. The co-integration of devices using GaN and 2D materials offers a promising alternative [136]. To increase the clarity of the general MEMS development process, the input, process, and output (IPO) components of this procedure are shown in Figure 18.

Traditional macro-sized devices are minimally impacted by the packaging they will eventually be shipped in. The ability to package MEMS for distribution once they are manufactured is a large hurdle because while MEMS can be batch-manufactured, the cost and difficulty of manufacturing the packaging is significant compared with that of the actual MEMS product [141]. The National Research Council states that understanding properties such as Coulomb friction, internal friction, solid–solid interface wear, and the influence of the chosen interface on reliability and performance are paramount to solving the challenges of packaging MEMS devices.

Packaging is also difficult because the packaging must be able to protect the device from ambient conditions but must also be able to provide controlled access to the environment. Because of the wide range of device types, there is no universal solution. Rao states that up to 90% of the total manufacturing costs of a device can derive from the fabrication of its packaging [142]. For example, a glucose-monitoring MEMS sensor is built at the micrometer scale; however, the packaging, including the casing and wires, is built at the centimeter scale (×10^4^ order of magnitude difference) [130].

#### 5.1.4. Additive Manufacturing

Three-dimensional printing falls under the category of additive manufacturing. This technology, which is similar to more conventional MEMS production techniques like surface micromachining, builds devices by layering on materials. In order to produce tiny 3D structures, inkjet printing spreads tiny droplets of colloidal material [49,143,144]. Fused deposition modeling (FDM) creates three-dimensional objects by melting polymers and depositing them along predetermined routes [145]. The feature sizes and maximum object sizes in 3D printing methods typically vary depending on a number of factors, such as the printing method, material, and printer type [146,147,148]. The final MEMs structure is affected by a number of parameters, including time, printing quality, and resolution [66]. MEMS fabrication via additive manufacturing makes it possible for complex 3D structures to be created, with a quick design process, allowing for customization and increased accuracy [145]. This improves structural MEMS applications in packaging, microelectronics, and microfluidics. Microfluidic devices, which are essential for artificial organs, can be quickly prototyped and produced in large quantities thanks to 3D printing. Miniaturized sensors can be placed to monitor bioceramic scaffolds that contain calcium phosphate apatite, providing vital sensory data for 3D-printed scaffolds [149,150,151,152]. By decreasing the stiffness constant of an electrostatically actuated cantilever-based MEMS switch, Kasambe et al. lowered the pull-in voltage while preserving important mechanical characteristics like stress and resonance frequency, they adjusted the cantilever’s size and shape using design optimization, mechanical modeling, 3D modeling, and Finite Element Method analysis to make sure it would continue to be strong and effective even when running at lower voltages [153].

Stereolithography, which cross-links polymer solutions using lasers, is frequently linked to 3D printing. Precision structuring and flexible 3D microfabrication are made possible in MEMS production through advanced techniques such as two-photon polymerization (2PP), selective laser sintering, continuous liquid interface production (CLIP), and projection microstereolithography (PμSL). These methods enable the production of intricate microfluidic devices with great precision and efficiency. They entail layer-by-layer particle fusion and the laser beam activation of photosensitive materials. Specifically, CLIP eliminates layer-by-layer restrictions, allowing for the continuous development of microstructures with high oxygen permeability [154]. Two-photon polymerization is better than projection microstereolithography for electrothermal microactuators, according to Ertugrul et al., since it can produce more complex, smaller, and non-symmetric structures [155].

Additive manufacturing techniques such as 3D printing are very desirable technologies, especially for prototyping novel devices. However, 3D-printed MEMS suffer from thermal shrinkage when printing polymer-based, green ceramic, and metal objects [66]. Blachowicz points out that the feature sizes are limited depending on the technology used, as seen in Table 2. The smallest feature size using 3D-printing methods is around 85 nm.

#### 5.1.5. Standardization

In the development of novel devices, the roles of design and fabrication are inseparable. If too much emphasis is placed on the design, without regard for the fabrication process, there may be no reasonable way to manufacture the end product. On the other hand, if all the focus is on the fabrication process, there is no room for imagination to create the unknown. Manufacturing techniques for MEMS devices still need to be standardized, allowing for reproducible mass production at lower costs [136]. While some strides have been made to standardize the fabrication of MEMS in commercial settings, Niekiel suggests that the research environments would also benefit from this practice [156].

Standardized methods for testing, characterization, and metrology are basic requirements for the development and manufacturing of novel MEMS devices [141]. The National Research Council also states that once this baseline is established, spreading this knowledge quickly and efficiently is foundational to the rapid progression of the MEMS field. One challenge to any newcomers to this field of study is the high level of knowledge about the fabrication process that is required before the design process can begin [142]. If the MEMS knowledge base can continue to grow and rapidly be dispersed, then more participants will be able to enter the field and begin working on advancing the latest technologies.

### 5.2. Prospective Research Trends

#### 5.2.1. AI Integrated Personalized Healthcare

MEMS devices that are capable of collecting real-time individual data and monitoring patient responses to treatments, enabling more tailored therapeutic approaches, could become a major contributor to personalized medicine. The majority of modern medicine is reactive, where medical providers diagnose the current illness based on past or present symptoms. Early intervention often has a large impact on the long-term outcome for the patient. Because artificial intelligence (AI) and [157,158,159,160] machine learning (ML) thrive [161] in a predictive environment, they could be powerful tools, using past or present symptom data to diagnose an illness even before its onset. Once a disease is diagnosed, MEMS, paired with AI using real-time data analysis, decision-making, and predictive modeling, could also help to optimize and implement the necessary treatment plan. In the future, various biomedical MEMS sensors could reside within the human body, and MEMS drug delivery systems could activate when needed.

#### 5.2.2. In Situ Energy Harvesting and Robotics

The foundational technology that will make personalized AI integrated healthcare possible is the ability to power micro- and nanoscale devices within the human body. There is a growing demand and interest in the field of in situ energy harvesting, where the basic concept is obtaining energy from the surrounding environment to power the given sensor or actuator. There are a few avenues currently being explored, such as piezoelectric-, thermoelectric-, and triboelectric-based devices. Piezoelectric devices use changes in the mechanical strain of the given piezoelectric material, which produce a measure of electricity. The concept of thermoelectric devices is similar; however, these devices use changes in temperature to induce electricity. Lastly, triboelectric devices seek to glean energy from the static electricity produced from the friction between two materials. This technology shows great potential, and one group has already developed two successfully self-powered devices based on triboelectricity [139,140]. Ultimately, these advances could lead to the development of microscale robots that can aid the human body by collecting data or actively moving to areas controlled by healthcare professionals or the individual.

#### 5.2.3. Lab-on-a-Chip

**Figure 19:**
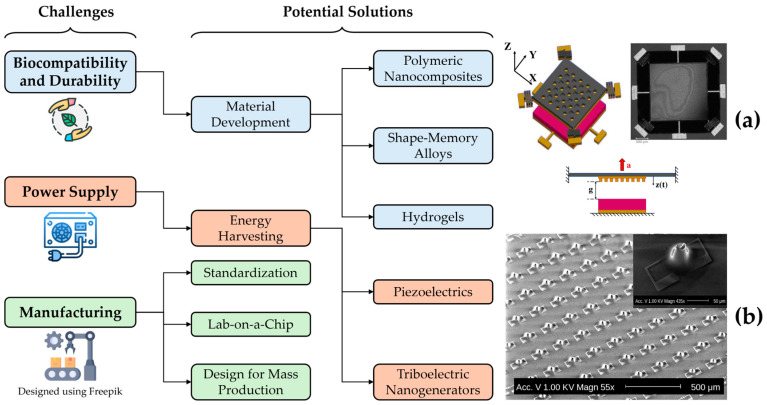
Current challenges and potential solutions for biomedical MEMS. (**a**) Self-powered triboelectric accelerometer (reprinted with permission from Elsevier) [139]; (**b**) mass production of Pluronic hydrogel-based MEMS devices (reprinted with permission from Elsevier) [163].

Lab-on-a-Chip (LOC) technology promotes the idea that one or more laboratory tasks can fit on a single microscale chip. Advances in LOC would be highly beneficial for the chemistry, biology, and medical fields. This technology can be used in chemical reactions by mixing fluids, as well as in medical diagnostics by taking small samples and placing them on the chip. This has implications for the vast amounts of lab work that is currently carried out in healthcare. Instead of requiring from milliliters to liters of blood or other bodily fluids, one drop may be sufficient to run all of the necessary tests [132,162]. Figure 19 provides an overlook of the current challenges facing the biomedical MEMS field, as well as a few of the potential solutions to these challenges.

## 6. Conclusions

Biomedical MEMS devices combine mechanical and electrical components at the micro- and nanoscales to provide high-performance, compact, and effective solutions for contemporary healthcare. The materials, classifications, and production procedures essential to the advancement of MEMS technology have been thoroughly covered in this review. MEMs manufacturing processes that are critical to the industries discussed in this paper are elaborated, along with their specific areas of application. The biomedical applications of MEMS in biosensors, actuators, microfluidics, and hybrid devices are discussed. The review addresses the limitations of biomedical applications, such as the necessity of material compatibility, power efficiency, and miniaturization. It describes the current challenges and potential solutions based on further developments in material science and fabrication methods, which will improve the integration and functionality of these devices. The gaps identified will enable researchers to concentrate on addressing unmet needs and advancing cutting-edge biomedical MEMS technology, ultimately facilitating the creation of more effective and innovative biomedical devices and enhancing patient care and outcomes.

## Figures and Tables

**Table 1:** Comparison of various MEMS manufacturing techniques.

Process	Bulk Micromachining [80]	Surface Micromachining [81]	LIGA [82]
**Main Process Type**	Subtractive	Additive	Additive/molding
**Workflow Sophistication**	Moderate	High	Very high
**Mass Production**	Moderate	Moderate	High
**Tailored for Metal Usage**	No	Yes	Yes
**Materials Used**	Silicon	Silicon and thin films	SU-8, Metals (Ni, Au, Cu)
**Manufacturing Cost**	Moderate	Moderate	High
**Accuracy and Quality**	Moderate	High	Very high
**Minimal Feature Size**	1 µm	100 nm to 1 µm	1–2 µm
**Dicing**	Blade dicing, laser dicing	Laser dicing, blade dicing	Laser dicing, diamond blade dicing
**Packaging Features**	Wafer-level packaging	Microfluidic packaging	Molded packaging

**Table 2:** MEMS 3D-printing technology resolutions [66].

Technology	Min. Feature Size	Material
Selective laser sintering	<400 μm	Polymers
Fused deposition modeling	200 μm	Polymers
Robot dispensing	200 μm	Hydrogels
Stereolithography	30–70 μm	Photosensitive Polymers
3D inkjet printing	28 μm	Photoresist
Resonant direct laser writing	1–4 μm	IP-Dip Photoresist
Multiphoton absorption polymerization	1 μm	SU8 Photoresist
Two-photon polymerization	0.28–1.5 μm	Photoresist
Direct laser writing	0.085–1.5 μm	Photoresist

## Data Availability

The data presented in this study are available on request from the correspondence author.

## References

[1] Łukasiak L., Jakubowski A. (2010). History of Semiconductors. J. Telecommun. Inf. Technol..

[2] Tai Y.-C., Zhou Z., Wang Z., Lin L. (2012). Introduction to MEMS. Microsystems and Nanotechnology.

[3] Hossain N., Mahmud M.Z.A., Hossain A., Rahman M.K., Islam M.S., Tasnim R., Mobarak M.H. (2024). Advances of Materials Science in MEMS Applications: A Review. Results Eng..

[4] Mohd Ghazali F.A., Hasan M.N., Rehman T., Nafea M., Mohamed Ali M.S., Takahata K. (2020). MEMS Actuators for Biomedical Applications: A Review. J. Micromech. Microengineering.

[5] Ke C., Pugno N., Peng B., Espinosa H. (2005). Experiments and Modeling of Carbon Nanotube-Based NEMS Devices. J. Mech. Phys. Solids.

[6] Bhushan B. (2007). Nanotribology and Nanomechanics of MEMS/NEMS and BioMEMS/BioNEMS Materials and Devices. Microelectron. Eng..

[7] Bhushan B., Bhushan B. (2004). Micro/Nanotribology of MEMS/NEMS Materials and Devices. Springer Handbook of Nanotechnology.

[8] Bhushan B. (2001). Fundamentals of Tribology and Bridging the Gap Between the Macro- and Micro/Nanoscales.

[9] Knüttel T. (2013). Laser Texturing of Surfaces in Thin-Film Silicon Photovoltaics-A Comparison of Potential Processes. J. Laser Micro Nanoeng..

[10] Zorman C.A., Parro R.J., Friedrichs P., Kimoto T., Ley L., Pensl G. (2009). Micro- and Nanomechanical Structures for Silicon Carbide MEMS and NEMS. Silicon Carbide.

[11] Mehregany M., Zorman C.A., Rajan N. (1998). Chien Hung Wu Silicon Carbide MEMS for Harsh Environments. Proc. IEEE.

[12] (2020). Handbook of Silicon Based MEMS Materials and Technologies.

[13] Kim B.J., Meng E. (2016). Review of Polymer MEMS Micromachining. J. Micromech. Microengineering.

[14] Khosla A., Gray B.L. (2012). (Invited) Micropatternable Multifunctional Nanocomposite Polymers for Flexible Soft NEMS and MEMS Applications. ECS Trans..

[15] Yunas J., Mulyanti B., Hamidah I., Mohd Said M., Pawinanto R.E., Wan Ali W.A.F., Subandi A., Hamzah A.A., Latif R., Yeop Majlis B. (2020). Polymer-Based MEMS Electromagnetic Actuator for Biomedical Application: A Review. Polymers.

[16] Tambe N.S., Bhushan B. (2005). Micro/Nanotribological Characterization of PDMS and PMMA Used for BioMEMS/NEMS Applications. Ultramicroscopy.

[17] Liu C. (2007). Recent Developments in Polymer MEMS. Adv. Mater..

[18] Alonzo S.M.M., Bentley J., Desai S., Bastakoti B.P. (2023). Hydrothermal Synthesis of Hierarchical Microstructure Tungsten Oxide/Carbon Nanocomposite for Supercapacitor Application. Sci. Rep..

[19] Banerjee A., Pandey S.S., Mastrangelo C.H. MEMS Stiction Supression with Sacrificial Polystyrene Nanoparticles. Proceedings of the 2017 IEEE SENSORS.

[20] Roussel M., Malhaire C., Deman A.-L., Chateaux J.-F., Petit L., Seveyrat L., Galineau J., Guiffard B., Seguineau C., Desmarres J.-M. (2014). Electromechanical Study of Polyurethane Films with Carbon Black Nanoparticles for MEMS Actuators. J. Micromech. Microengineering.

[21] Reed H.A., White C.E., Rao V., Allen S.A.B., Henderson C.L., Kohl P.A. (2001). Fabrication of Microchannels Using Polycarbonates as Sacrificial Materials. J. Micromech. Microengineer..

[22] Hangarter C.M., George T., Myung N.V., Osaka T., Datta M., Shacham-Diamand Y. (2010). Electrochemically Fabricated Microelectromechanical Systems/Nanoelectromechanical Systems (MEMS/NEMS). Electrochemical Nanotechnologies.

[23] Panda A. (2003). Electrodeposition of Nickel-Copper Alloys and Nickel-Copper-Alumina Nanocomposites into Deep Recesses for MEMS. Ph.D. Thesis.

[24] Phatthanakun R., Deekla P., Pummara W., Sriphung C., Pantong C., Chomnawang N. Fabrication and Control of Thin-Film Aluminum Microheater and Nickel Temperature Sensor. Proceedings of the 8th Electrical Engineering/Electronics, Computer, Telecommunications and Information Technology (ECTI) Association of Thailand-Conference 2011.

[25] Hu T., Fang K., Zhang Z., Jiang X., Zhao Y. (2019). The Hybrid Fabrication Process of Metal/Silicon Composite Structure for MEMS S&A Device. Micromachines.

[26] Muralt P. (2008). Recent Progress in Materials Issues for Piezoelectric MEMS. J. Am. Ceram. Soc..

[27] Desai S., Lovell M. (2012). Modeling Fluid–Structure Interaction in a Direct Write Manufacturing Process. J. Mater. Process. Technol..

[28] Desai S., Lovell M., Cordle J. (2007). Coupled Field Analysis of a Piezoelectric Bimorph Disc in a Direct Write Process. Compos. Part B Eng..

[29] Desai S., Lovell M. (2009). Computational Fluid Dynamics Analysis of a Direct Write Manufacturing Process. Int. J. Nanomanuf..

[30] Wasa K., Matsushima T., Adachi H., Kanno I., Kotera H. (2012). Thin-Film Piezoelectric Materials For a Better Energy Harvesting MEMS. J. Microelectromech. Syst..

[31] Eom C.-B., Trolier-McKinstry S. (2012). Thin-Film Piezoelectric MEMS. MRS Bull..

[32] Torkashvand Z., Shayeganfar F., Ramazani A. (2024). Nanomaterials Based Micro/Nanoelectromechanical System (MEMS and NEMS) Devices. Micromachines.

[33] Li X., Sun M., Shan C., Chen Q., Wei X. (2018). Mechanical Properties of 2D Materials Studied by In Situ Microscopy Techniques. Adv. Mater. Interfaces.

[34] Ferrari P.F., Kim S., Van Der Zande A.M. (2023). Nanoelectromechanical Systems from Two-Dimensional Materials. Appl. Phys. Rev..

[35] Geim A.K. (2009). Graphene: Status and Prospects. Science.

[36] Fan X., He C., Ding J., Gao Q., Ma H., Lemme M.C., Zhang W. (2024). Graphene MEMS and NEMS 2024. Microsyst. Nanoeng..

[37] Kommanaboina N.M. (2023). Development of MEMS-Based Devices for Characterizing 2D Nanomaterials at Low Temperatures. Ph.D. Thesis.

[38] Liu X., Mwangi M., Li X., O’Brien M., Whitesides G.M. (2011). Paper-Based Piezoresistive MEMS Sensors. Lab. Chip.

[39] Eddy D.S., Sparks D.R. (1998). Application of MEMS Technology in Automotive Sensors and Actuators. Proc. IEEE.

[40] Zhu J., Liu X., Shi Q., He T., Sun Z., Guo X., Liu W., Sulaiman O.B., Dong B., Lee C. (2019). Development Trends and Perspectives of Future Sensors and MEMS/NEMS. Micromachines.

[41] Du H., Chau F., Zhou G. (2016). Mechanically-Tunable Photonic Devices with On-Chip Integrated MEMS/NEMS Actuators. Micromachines.

[42] Ouakad H.M. (2017). Comprehensive Numerical Modeling of the Nonlinear Structural Behavior of MEMS/NEMS Electrostatic Actuators under the Effect of the van Der Waals Forces. Microsyst. Technol..

[43] Meng F., Zhang C., Zhang G., Renxin W., He C., Yang Y., Cui J., Zhang W., Jia L. (2025). A Novel AlN/Sc0. 2Al0.8N-Based Piezoelectric Composite Thin-Film-Enabled Bioinspired Honeycomb MEMS Hydrophone.

[44] Islam N. (2012). Microelectromechanical Systems and Devices.

[45] Vyawahare S., Griffiths A.D., Merten C.A. (2010). Miniaturization and Parallelization of Biological and Chemical Assays in Microfluidic Devices. Chem. Biol..

[46] Desai S., Perkins J., Harrison B.S., Sankar J. (2010). Understanding Release Kinetics of Biopolymer Drug Delivery Microcapsules for Biomedical Applications. Mater. Sci. Eng. B.

[47] Aldawood F.K., Parupelli S.K., Andar A., Desai S. (2024). 3D Printing of Biodegradable Polymeric Microneedles for Transdermal Drug Delivery Applications. Pharmaceutics.

[48] Perkins J., Yi H., Ye S.H., Wagner W., Desai S. (2014). Direct Write Manufacturing of Controlled Release Coatings for Drug Eluting Cardiovascular Stents. J. Bio. Med. Res. Part A.

[49] Adarkwa E., Roy A., Ohodnicki J., Lee B., Kumta P.N., Desai S. (2023). 3D Printing of Drug-Eluting Bioactive Multifunctional Coatings for Orthopedic Applications. Int. J. Bioprinting.

[50] Lin L., Chung C.-K. (2021). PDMS Microfabrication and Design for Microfluidics and Sustainable Energy Application: Review. Micromachines.

[51] Madou M.J. (2018). Fundamentals of Microfabrication: The Science of Miniaturization.

[52] Hilbert J.L. (2008). RF-MEMS for Wireless Communications. IEEE Commun. Mag..

[53] Shao B., Lu C., Xiang Y., Li F., Song M. (2024). Comprehensive Review of RF MEMS Switches in Satellite Communications. Sensors.

[54] Will-Cole A.R., Hassanien A.E., Calisgan S.D., Jeong M.-G., Liang X., Kang S., Rajaram V., Martos-Repath I., Chen H., Risso A. (2022). Tutorial: Piezoelectric and Magnetoelectric N/MEMS—Materials, Devices, and Applications. J. Appl. Phys..

[55] Luo B., Will-Cole A.R., Dong C., He Y., Liu X., Lin H., Huang R., Shi X., McConney M., Page M. (2024). Magnetoelectric Microelectromechanical and Nanoelectromechanical Systems for the IoT. Nat. Rev. Electr. Eng..

[56] Safaei M., Sodano H.A., Anton S.R. (2019). A Review of Energy Harvesting Using Piezoelectric Materials: State-of-the-Art a Decade Later (2008–2018). Smart Mater. Struct..

[57] Gu L. (2011). Low-Frequency Piezoelectric Energy Harvesting Prototype Suitable for the MEMS Implementation. Microelectron. J..

[58] Huang M., Hou C., Li Y., Liu H., Wang F., Chen T., Yang Z., Tang G., Sun L. (2019). A Low-Frequency MEMS Piezoelectric Energy Harvesting System Based on Frequency Up-Conversion Mechanism. Micromachines.

[59] Bhushan B. (2010). Springer Handbook of Nanotechnology.

[60] Solgaard O., Godil A.A., Howe R.T., Lee L.P., Peter Y.-A., Zappe H. (2014). Optical MEMS: From Micromirrors to Complex Systems. J. Microelectromech. Syst..

[61] Tsai C., Tsai J. (2015). MEMS Optical Switches and Interconnects. Displays.

[62] Wu M.C., Solgaard O., Ford J.E. (2006). Optical MEMS for Lightwave Communication. J. Light Technol..

[63] Ma X., Kuo G.-S. (2003). Optical Switching Technology Comparison: Optical Mems vs. Other Technologies. IEEE Commun. Mag..

[64] Misrak A., Chauhan T., Bhandari R., Chowdhury A.R., Lakshminarayana A., Mirza F., Bazehhour B.G., Vujosevic M., Agonafer D. (2021). Impact of Die Attach Sample Preparation on Its Measured Mechanical Properties for MEMS Sensor Applications. J. Microelectron. Electron. Packag..

[65] Mohammed A.A., Moussa W.A., Lou E. (2008). High Sensitivity MEMS Strain Sensor: Design and Simulation. Sensors.

[66] Blachowicz T., Ehrmann A. (2020). 3D Printed MEMS Technology—Recent Developments and Applications. Micromachines.

[67] Abbasi R., Mayyas M., Ghasemian M.B., Centurion F., Yang J., Saborio M., Allioux F.-M., Han J., Tang J., Christoe M.J. (2020). Photolithography–Enabled Direct Patterning of Liquid Metals. J. Mater. Chem. C.

[68] Barajas-Valdes U., Suárez O.M. (2020). Nanomechanical Properties of Thin Films Manufactured via Magnetron Sputtering from Pure Aluminum and Aluminum-Boron Targets. Thin Solid Film..

[69] Davranche M., Bollinger J.-C. (2000). Release of Metals from Iron Oxyhydroxides under Reductive Conditions: Effect of Metal/Solid Interactions. J. Colloid Interface Sci..

[70] Salehi M., Heidari P., Ruhani B., Kheradmand A., Purcar V., Căprărescu S. (2021). Theoretical and Experimental Analysis of Surface Roughness and Adhesion Forces of MEMS Surfaces Using a Novel Method for Making a Compound Sputtering Target. Coatings.

[71] Mo Z., Wang F., Li J., Liu Q., Zhang G., Li W., Yang C., Sun R. (2023). Temporary Bonding and Debonding in Advanced Packaging: Recent Progress and Applications. Electronics.

[72] Pal P., Sato K., Gosalvez M.A., Shikida M. (2007). Study of Rounded Concave and Sharp Edge Convex Corners Undercutting in CMOS Compatible Anisotropic Etchants. J. Micromech. Microengineer..

[73] Pal P., Sato K. (2010). Fabrication Methods Based on Wet Etching Process for the Realization of Silicon MEMS Structures with New Shapes. Microsyst. Technol..

[74] De Boer M.J., Gardeniers J.G.E., Jansen H.V., Smulders E., Gilde M.-J., Roelofs G., Sasserath J.N., Elwenspoek M. (2002). Guidelines for Etching Silicon MEMS Structures Using Fluorine High-Density Plasmas at Cryogenic Temperatures. J. Microelectromechanical Syst..

[75] Li H., Ruan Y., You Z., Song Z. (2020). Design and Fabrication of a Novel MEMS Relay with Low Actuation Voltage. Micromachines.

[76] Cao T., Hu T., Zhao Y. (2020). Research Status and Development Trend of MEMS Switches: A Review. Micromachines.

[77] Fogel O., Winter S., Benjamin E., Krylov S., Kotler Z., Zalevsky Z. (2018). 3D printing of functional metallic microstructures and its implementation in electrothermal actuators. Addit. Manuf..

[78] Zhang Y.-F., Cui M., Wu D.-P. (2023). Design and Fabrication of a MEMS Bandpass Filter with Different Center Frequency of 8.5–12 GHz. Micromachines.

[79] Lee S., Park S., Cho D.-I. (1999). The Surface/Bulk Micromachining (SBM) Process: A New Method for Fabricating Released MEMS in Single Crystal Silicon. J. Microelectromechanical Syst..

[80] Kauzlarick D. (2003). Fundamentals of Microfabrication, the Science of Miniaturization, 2nd Edition [Book Review]. IEEE Eng. Med. Biol. Mag..

[81] Nguyen N.-T., Hejazian M., Ooi C., Kashaninejad N. (2017). Recent Advances and Future Perspectives on Microfluidic Liquid Handling. Micromachines.

[82] Wang W., Soper S.A. (2006). Bio-MEMS: Technologies and Applications.

[83] Parupelli S.K., Desai S. (2023). The 3D Printing of Nanocomposites for Wearable Biosensors: Recent Advances, Challenges, and Prospects. Bioengineering.

[84] Desai S., Bidanda B., Bártolo P.J. (2021). Emerging Trends in the Applications of Metallic and Ceramic Biomaterials. Bio-Materials and Prototyping Applications in Medicine.

[85] Desai S., Shankar M.R. (2021). Emerging Trends in Polymers, Composites, and Nano Biomaterial Applications. Bio-Materials and Prototyping Applications in Medicine.

[86] Hajare R., Reddy V., Srikanth R. (2022). MEMS Based Sensors–A Comprehensive Review of Commonly Used Fabrication Techniques. Mater. Today Proc..

[87] Nazir S., Kwon O.S. (2022). Micro-Electromechanical Systems-Based Sensors and Their Applications. Appl. Sci. Converg. Technol..

[88] Varney M.W., Aslam D.M., Janoudi A., Chan H.-Y., Wang D.H. (2011). Polycrystalline-Diamond MEMS Biosensors Including Neural Microelectrode-Arrays. Biosensors.

[89] Yunus N.A.M., Halin I.A., Sulaiman N., Ismail N.F., Sheng O.K. (2015). Valuation on MEMS Pressure Sensors and Device Applications. Int. J. Electron. Commun. Eng..

[90] Lakshmai G.S., Rao K.S., Sravani K.G. (2023). Design and Analysis of MEMS Pressure Sensor Based on Various Principles of Microcantilever Beam. IEEE Trans. NanoBiosci..

[91] Kolluri S.S.K., Durai S.A. (2024). Wearable Micro-electro-mechanical Systems Pressure Sensors in Health Care: Advancements and Trends—A Review. IET Wirel. Sens. Syst..

[92] Rohan R., Venkadeshwaran K. Measurement of Human Blood Pressure Using Mems Pressure Sensor. Proceedings of the 2022 9th International Conference on Computing for Sustainable Global Development (INDIACom).

[93] Chattopadhyay M., Chowdhury D. (2017). Design and Performance Analysis of MEMS Capacitive Pressure Sensor Array for Measurement of Heart Rate. Microsyst. Technol..

[94] Kalvesten E., Smith L., Tenerz L., Stemme G. The First Surface Micromachined Pressure Sensor for Cardiovascular Pressure Measurements. Proceedings of the Proceedings MEMS 98. IEEE. Eleventh Annual International Workshop on Micro Electro Mechanical Systems. An Investigation of Micro Structures, Sensors, Actuators, Machines and Systems (Cat. No. 98CH36176).

[95] Meena K.V., Sankar A.R. (2021). Biomedical Catheters with Integrated Miniature Piezoresistive Pressure Sensors: A Review. IEEE Sens. J..

[96] Pandey Y., Singh S.P. (2023). Recent Advances in Bio-MEMS and Future Possibilities: An Overview. J. Inst. Eng. India Ser. B.

[97] Abdul B. (2023). Development of a Novel Silicon Membrane MEMS Capacitive Pressure Sensor for Biological Applications. Eng. Proc..

[98] Chircov C., Grumezescu A.M. (2022). Microelectromechanical Systems (MEMS) for Biomedical Applications. Micromachines.

[99] Vante A.B., Kanish T.C. (2024). Fluid-Structure Interaction and Experimental Studies of Passive Check Valve Based Piezoelectric Micropump for Biomedical Applications. Adv. Mater. Process. Technol..

[100] He J.-H., He C.-H., Qian M.-Y., Alsolami A.A. (2024). Piezoelectric Biosensor Based on Ultrasensitive MEMS System. Sens. Actuators Phys..

[101] Wu N., Tian Y., Zou X., Zhai Y., Barringhaus K., Wang X. (2013). A Miniature Fiber Optic Blood Pressure Sensor and Its Application in in Vivo Blood Pressure Measurements of a Swine Model. Sens. Actuators B Chem..

[102] Young D.J. Interface Electronics for MEMS-Based Wireless Sensing Applications. Proceedings of the 2010 International Symposium on VLSI Design, Automation and Test.

[103] Kulkarni M.B., Ayachit N.H., Aminabhavi T.M. (2022). Biosensors and Microfluidic Biosensors: From Fabrication to Application. Biosensors.

[104] Helvajian H. (1999). Microengineering Aerospace Systems.

[105] Belyustin A.A. (2011). The Centenary of Glass Electrode: From Max Cremer to F. G. K. Baucke. J. Solid State Electrochem..

[106] Clark L.C., Wolf R., Granger D., Taylor Z. (1953). Continuous Recording of Blood Oxygen Tensions by Polarography. J. Appl. Physiol..

[107] Barlian A.A., Park W.-T., Mallon J.R., Rastegar A.J., Pruitt B.L. (2009). Review: Semiconductor Piezoresistance for Microsystems. Proc. IEEE.

[108] Gatzen H.H., Saile V., Leuthold J. (2015). Introduction—MEMS, a Historical Perspective. Micro and Nano Fabrication.

[109] Sander C.S., Knutti J.W., Meindl J.D. (1980). A Monolithic Capacitive Pressure Sensor with Pulse-Period Output. IEEE Trans. Electron Devices.

[110] Michaels J.E., Michaels T.E. (2005). Detection of Structural Damage from the Local Temporal Coherence of Diffuse Ultrasonic Signals. IEEE Trans. Ultrason. Ferroelectr. Freq. Control.

[111] Kaisti M., Panula T., Leppänen J., Punkkinen R., Tadi M.J., Vasankari T., Jaakkola S., Kiviniemi T., Airaksinen J., Kostiainen P. (2019). Clinical Assessment of a Non-Invasive Wearable MEMS Pressure Sensor Array for Monitoring of Arterial Pulse Waveform, Heart Rate and Detection of Atrial Fibrillation. NPJ Digit. Med..

[112] Al-Qatatsheh A., Morsi Y., Zavabeti A., Zolfagharian A., Salim N., Kouzani A.Z., Mosadegh B., Gharaie S. (2020). Blood Pressure Sensors: Materials, Fabrication Methods, Performance Evaluations and Future Perspectives. Sensors.

[113] Polat E.O., Cetin M.M., Tabak A.F., Güven E.B., Uysal B.Ö., Arsan T., Kabbani A., Hamed H., Gül S.B. (2022). Transducer Technologies for Biosensors and Their Wearable Applications. Biosensors.

[114] Eidi A. (2023). Design and Evaluation of an Implantable MEMS Based Biosensor for Blood Analysis and Real-Time Measurement. Microsyst. Technol..

[115] Lakshmi G.S., Karumuri S.R., Kondavitee G.S., Lay-Ekuakille A. (2023). Design and Performance Analysis of a Microbridge and Microcantilever-Based MEMS Pressure Sensor for Glucose Monitoring. IEEE Sens. J..

[116] Gemelli A., Tambussi M., Fusetto S., Aprile A., Moisello E., Bonizzoni E., Malcovati P. (2023). Recent Trends in Structures and Interfaces of MEMS Transducers for Audio Applications: A Review. Micromachines.

[117] Hamed H., Eldiasty M., Seyedi-Sahebari S.-M., Abou-Ziki J.D. (2023). Applications, Materials, and Fabrication of Micro Glass Parts and Devices: An Overview. Mater. Today.

[118] Cugno M. (2022). Light Actuated Microvalve. Ph.D. Thesis.

[119] Richter M., Anheuer D., Wille A., Congar Y., Wackerle M. (2023). Multistage Micropump System towards Vacuum Pressure. Actuators.

[120] Ficai D., Gheorghe M., Dolete G., Mihailescu B., Svasta P., Ficai A., Constantinescu G., Andronescu E. (2022). Microelectromechanical Systems Based on Magnetic Polymer Films. Micromachines.

[121] Matsubara T., Choi J.S., Kim D.-H., Kim J. (2022). A Microfabricated Pistonless Syringe Pump Driven by Electro-Conjugate Fluid with Leakless On/Off Microvalves. Small.

[122] Li B., Zhang L., Bai S., Jin J., Chen H. (2024). A Brief Overview of Passive Microvalves in Microfluidics: Mechanism, Manufacturing, and Applications. Biomicrofluidics.

[123] Gavali S.R., Pawar P.M. (2024). Computational Analysis of a Four-Flap Valveless Micropump (FFVM) for Low Reynolds Number Applications in Microfluidic Systems. Phys. Scr..

[124] Khadanga S. (2021). Fabrication of MEMS Pressure Sensor on Thin Film Membrane. Engpaper J..

[125] Vyas S., Alhussainy A.K., Raju Y.K., Manjunatha M., Srivastava A.P., Jain A., Vijetha T. (2024). Analytical Review on Enhancing Sustainability in Microsystems by Integrating MEMS for Compact Design. E3S Web Conf. EDP Sci..

[126] Arnoult T., Leclercq C., Ghouila-Houri C., Mazzamurro A., Viard R., Garnier E., Poussot-Vassal C., Merlen A., Sipp D., Pernod P. (2023). Subsonic Cavity Flow Control with Micro-Magneto-Mechanical Systems (MMMS) Microvalves. Sens. Actuators Phys..

[127] Morkvenaite-Vilkonciene I., Bucinskas V., Subaciute-Zemaitiene J., Sutinys E., Virzonis D., Dzedzickis A. (2022). Development of Electrostatic Microactuators: 5-Year Progress in Modeling, Design, and Applications. Micromachines.

[128] Sharma S., Kohli N., Brière J., Nabki F., Ménard M. (2022). Integrated 1× 3 MEMS Silicon Nitride Photonics Switch. Opt. Express.

[129] Aishan Y., Funano S., Sato A., Ito Y., Ota N., Yalikun Y., Tanaka Y. (2022). Bio-Actuated Microvalve in Microfluidics Using Sensing and Actuating Function of Mimosa Pudica. Sci. Rep..

[130] Huang X., Li S., Schultz J.S., Wang Q., Lin Q. (2009). A MEMS Affinity Glucose Sensor Using a Biocompatible Glucose-Responsive Polymer. Sens. Actuators B Chem..

[131] Ni M., Tong W.H., Choudhury D., Rahim N.A.A., Iliescu C., Yu H. (2009). Cell Culture on MEMS Platforms: A Review. Int. J. Mol. Sci..

[132] Christensen T.B., Pedersen C.M., Gröndahl K.G., Jensen T.G., Sekulovic A., Bang D.D., Wolff A. (2007). PCR Biocompatibility of Lab-on-a-Chip and MEMS Materials. J. Micromech. Microengineering.

[133] Voskerician G., Shive M.S., Shawgo R.S., Recum H.V., Anderson J.M., Cima M.J., Langer R. (2003). Biocompatibility and Biofouling of MEMS Drug Delivery Devices. Biomaterials.

[134] Kotzar G., Freas M., Abel P., Fleischman A., Roy S., Zorman C., Moran J.M., Melzak J. (2002). Evaluation of MEMS Materials of Construction for Implantable Medical Devices. Biomaterials.

[135] Hopcroft M.A., Nix W.D., Kenny T.W. (2010). What Is the Young’s Modulus of Silicon?. J. Microelectromechanical Syst..

[136] Villanueva L.G., Bausells J., Brugger J. (2016). Grand Challenge in N/MEMS. Front. Mech. Eng..

[137] Ionov L. (2014). Hydrogel-Based Actuators: Possibilities and Limitations. Mater. Today.

[138] Medical Devices–Angela N Johnson. https://angelanjohnson.com/medicaldevices/.

[139] Alzgool M., Tian Y., Davaji B., Towfighian S. (2023). Self-Powered Triboelectric MEMS Accelerometer. Nano Energy.

[140] Mousavi M., Alzgool M., Davaji B., Towfighian S. (2023). Event-Driven MEMS Vibration Sensor: Integration of Triboelectric Nanogenerator and Low-Frequency Switch. Mech. Syst. Signal Process..

[141] National R.C. (1997). Microelectromechanical Systems: Advanced Materials and Fabrication Methods.

[142] Rao R. (2022). An Introduction to MEMS (Micro-Electromechanical Systems). Prime Faraday Technol. Watch.

[143] Adarkwa E., Kotoka R., Desai S. (2021). 3D Printing of Polymeric Coatings on AZ31 Mg Alloy Substrate for Corrosion Protection of Biomedical Implants. Med. Devices Sens..

[144] Kumar_Parupelli S., Saudi S., Bhattarai N., Desai S. (2023). 3D Printing of PCL-Ceramic Composite Scaffolds for Bone Tissue Engineering Applications. Int. J. Bioprinting.

[145] Parupelli S.K., Desai S. (2020). Hybrid Additive Manufacturing (3D Printing) and Characterization of Functionally Gradient Materials via in Situ Laser Curing. Int. J. Adv. Manuf. Technol..

[146] Ogunsanya M., Isichei J., Desai S. (2023). Grid Search Hyperparameter Tuning in Additive Manufacturing Processes. Manuf. Lett..

[147] Yang M., Parupelli S.K., Xu Z., Desai S. (2024). Understanding the Effect of Dispersant Rheology and Binder Decomposition on 3D Printing of a Solid Oxide Fuel Cell. Micromachines.

[148] Yang M., Parupelli S.K., Xu Z., Desai S. (2024). Three-Dimensional-Printed Composite Structures: The Effect of LSCF Slurry Solid Loading, Binder, and Direct-Write Process Parameters. Materials.

[149] Marquetti I., Desai S. (2018). Molecular Modeling the Adsorption Behavior of Bone Morphogenetic Protein-2 on Hydrophobic and Hydrophilic Substrates. Chem. Phys. Lett..

[150] Marquetti I., Desai S. (2022). Nanoscale Topographical Effects on the Adsorption Behavior of Bone Morphogenetic Protein-2 on Graphite. Int. J. Mol. Sci..

[151] Marquetti I., Desai S. (2019). Orientation Effects on the Nanoscale Adsorption Behavior of Bone Morphogenetic Protein-2 on Hydrophilic Silicon Dioxide. RSC Adv..

[152] Plander I., Stepanovsky M. (2016). MEMS Optical Switch: Switching Time Reduction. Open Comput. Sci..

[153] Kasambe P.V., Bhole K.S., Raykar N.R., Oza A.D., Ramesh R., Bhoir D.V. (2022). Mechanical Modeling, Numerical Investigation and Design of Cantilever Beam for Low Pull-in MEMS Switch. Int. J. Interact. Des. Manuf. IJIDeM.

[154] Yazdanpanah Z., Johnston J.D., Cooper D.M., Chen X. (2022). 3D Bioprinted Scaffolds for Bone Tissue Engineering: State-of-the-Art and Emerging Technologies. Front. Bioeng. Biotechnol..

[155] Ertugrul I., Akkus N., Yuce H. (2019). Fabrication of MEMS-based electrothermal microactuators with additive manufacturing technologies. Mater. Tehnol..

[156] Niekiel M.F., Meyer J.M., Lewitz H., Kittmann A., Nowak M.A., Lofink F., Meyners D., Zollondz J.-H. (2023). What MEMS Research and Development Can Learn from a Production Environment. Sensors.

[157] Almakaeel H., Albalawi A., Desai S. (2018). Artificial Neural Network Based Framework for Cyber Nano Manufacturing. Manuf. Lett..

[158] Nandipati M., Fatoki O., Desai S. (2024). Bridging Nanomanufacturing and Artificial Intelligence—A Comprehensive Review. Materials.

[159] Elhoone H., Zhang T., Anwar M., Desai S. (2020). Cyber-Based Design for Additive Manufacturing Using Artificial Neural Networks for Industry 4.0. Int. J. Prod. Res..

[160] Akter T., Desai S. (2018). Developing a Predictive Model for Nanoimprint Lithography Using Artificial Neural Networks. Mater. Des..

[161] Nandipati M., Ogunsanya M., Desai S. (2024). Predictive Models for 3D Inkjet Material Printer Using Automated Image Analysis and Machine Learning Algorithms. Manuf. Lett..

[162] Verpoorte E., De Rooij N.F. (2003). Microfluidics Meets MEMS. Proc. IEEE.

[163] Guan T., Godts F., Ceyssens F., Vanderleyden E., Adesanya K., Dubruel P., Neves H.P., Puers R. (2012). Development and Fabrication of a Novel Photopatternable Electric Responsive Pluronic Hydrogel for MEMS Applications. Sens. Actuators Phys..

